# Therapeutic Reprogramming of Glioblastoma Phenotypic States Using Multifunctional Heparin Nanoparticles

**DOI:** 10.1002/advs.202509590

**Published:** 2025-11-03

**Authors:** Vadim Le Joncour, Austin D. Evans, Camilla Karoliina Bergström, Vignesh Kumar Rangasami, Sumanta Samanta, Nithiyanandan Krishnan, Yuji Teramura, Bo Nilsson, Patricia Hedenqvist, Elin Manell, Jons Hilborn, Marianne‐Jensen Waern, Oommen P. Varghese, Pirjo Laakkonen, Oommen P. Oommen

**Affiliations:** ^1^ Neuroscience Center Helsinki Institute of Life Science and iCAN Digital Precision Medicine Flagship Program University of Helsinki Haartmaninkatu 8 Helsinki 00290 Finland; ^2^ Bioengineering and Nanomedicine Lab Faculty of Medicine and Health Technology Tampere University Korkeakoulunkatu 3 Tampere 33720 Finland; ^3^ Department of Bioengineering Phil and Penny Knight Campus for Accelerating Scientific Impact University of Oregon Eugene OR 97403 USA; ^4^ Translational Chemical Biology Group Division of Macromolecular Chemistry Department of Chemistry‐Ångström Laboratory Uppsala University Uppsala SE75121 Sweden; ^5^ Cellular and Molecular Biotechnology Research Institute (CMB) National Institute of Advanced Industrial Science and Technology (AIST) AIST Tsukuba Central 5, 111 Higashi Tsukuba Ibaraki 3058565 Japan; ^6^ Department of Immunology Genetics and Pathology (IGP) Uppsala University Dag Hammarskjölds väg 20 Uppsala 75185 Sweden; ^7^ Department of Clinical Sciences Swedish University of Agricultural Sciences Uppsala 75105 Sweden; ^8^ Translational Cancer Medicine Research Program Faculty of Medicine University of Helsinki Helsinki 00010 Finland; ^9^ School of Pharmacy and Pharmaceutical Sciences Cardiff University King Edward VII Avenue Cardiff CF10 3NB UK

**Keywords:** blood‐brain barrier, drug delivery, glioma stem cells, heparin, nanoparticles

## Abstract

Glioblastomas (GB) are the most common and deadly primary malignant brain tumors due to their infiltrative growth and resistance to conventional therapies. GB cell plasticity and differentiation into drug‐resistant mesenchymal‐like (MES) states protect tumors from conventional treatments. This study introduces a novel precision medicine approach employing heparin‐based nanoparticles (HP‐NPs) engineered to cross the blood‐brain barrier and target MES‐like glioma stem cells (GSCs). Encapsulating doxorubicin (DOX) in HP‐NPs reduces drug‐mediated complement and coagulation cascades, enhancing hemocompatibility in human whole blood. In vitro, HP‐NPs demonstrate efficient uptake by patient‐derived GSCs. Preclinical evaluations in patient avatars indicate plain HP‐NPs outperform DOX‐loaded HP‐NPs in reducing GB progression. Transcriptomic studies show HP‐NPs downregulate heparin‐binding epidermal growth factor (HBEGF), shifting MES GSCs into less plastic astroglial‐like cells, impairing tumorigenesis. HP‐NPs are well‐tolerated and safe at therapeutic doses in healthy rats, offering a promising new paradigm in anticancer therapy to overcome GB recurrence and improve therapeutic outcomes.

## Graphical abstract

This study presents heparin‐derived nanoparticles (HP‐NPs) as a novel precision medicine platform that combines therapeutic and delivery functions. HP‐NPs target drug‐resistant glioblastoma stem cells, reprogramming them into a drug‐sensitive phenotype and significantly reducing tumor progression. By avoiding conventional cytotoxic drugs, HP‐NPs achieve microenvironmental reprogramming, offering a transformative, safer, and more effective therapeutic strategy for glioblastoma.

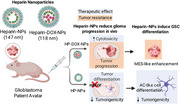

## 1 Introduction

Glioblastoma (GB) exhibits the highest incidence and worst prognosis, with poor survival rates despite intensive therapy. GB's fast progression is fueled by diverse cell phenotypes, including mesenchymal (MES)‐like, astrocyte (AC)‐like, oligodendrocyte precursor cell (OPC)‐like, and neuron precursor cell (NPC)‐like groups.^[^ ^1^ ^]^ Each cell state specializes in either proliferation, treatment resistance, or integration with healthy brain tissue, enabling therapy escape and lack of improvement on patient outcomes.^[^ ^2^ ^]^ Additional therapeutic challenges include the blood‐brain barrier (BBB) sheltering effect,^[^ ^3^ ^]^ immunosuppressive tumor microenvironment,^[^ ^4^ ^]^ and GB connectivity to neuronal networks.^[^ ^5^ ^]^ GB cell plasticity, crucial for tumor progression and recurrence, allows adaptation to therapies and environmental changes.^[^ ^6^ ^]^ Innate immune cells interact with GB cells within the GB tumor microenvironment and initiate NPC‐to‐MES transition conferring therapeutic resistance.^[^ ^7^ ^]^ MES‐like cells overexpressing epidermal growth factor receptor (EGFR), are notable drivers of therapeutic resistance and relapse, and their number has been shown to increase after chemotherapy and radiation.^[^ ^8^ ^]^ Currently, there are no clinical strategies to manipulate phenotypic GB transition states or to specifically eliminate MES‐like cells.

In an attempt to tackle GB progression, precision medicine targeting tumor vasculature (Bevacizumab vascular endothelial growth factor/VEGF scavenger antibody), tumor recurrence (Imatinib, platelet derived growth factor receptor/PDGFR inhibitor) or mitotic stromal and neoplastic cells (Enzastaurin, cytostatic and antiangiogenic), have been introduced to the clinics as monotherapy or in combination with a secondary agent in GB clinical trials.^[^ ^8^, ^9^ ^]^ However, none of these attempts have yielded significant improvement of the progression‐free patient survival. Indeed, none of these therapies target or affect GB stem cells (GSCs), which constitute a reservoir of chemo‐resistant cells fueling tumor relapse.^[^ ^6^ ^]^

Herein, we show that heparin (HP) derived nanocarriers (HP‐NPs) modulate multiple dimensions of GB progression and favorably alter plasticity of GB cells. HP is not BBB permeable unless reduced to its low molecular weight form (LMWH).^[^ ^10^ ^]^ However, HP is known for its anticoagulant activity and is used clinically for treating various thrombotic conditions such as thrombosis, pulmonary emboli, acute coronary syndromes, and ischemic cerebrovascular events.^[^ ^11^ ^]^ HP is also clinically implemented for treating pathologies such as asthma, inflammatory bowel disease, acute coronary syndrome, injury treatment, cystic fibrosis, and allergic rhinitis.^[^ ^12^ ^]^ HP possesses anti‐cancer properties^[^ ^13^ ^]^ and prevents metastatic progression.^[^ ^14^ ^]^ HP reprograms the tumor microenvironment by extenuating angiogenesis and tumor cell adhesion via inhibition of P‐selectin.^[^ ^15^ ^]^ HP has high affinity for P‐selectin,^[^ ^15^, ^16^ ^]^ an oncoprotein promoting GB progression and perturbing microglia and macrophage activation.^[^ ^17^ ^]^ Further, HP's antimetastatic effects^[^ ^14^ ^]^ is mediated by blocking P‐selectin‐dependent tumor‐cell–platelet interactions in blood‐borne metastasis.^[^ ^18^ ^]^ In glycocalyx injury models, intravenous HP restored BBB integrity.^[^ ^19^ ^]^ Recently, engineered lactoferrin‐heparin conjugates administered orally have been shown to slow down intracranial GB progression in xenograft models.^[^ ^20^ ^]^

We hypothesize that HP‐NPs could target heparin binding EGF (HBEGF), which interacts with EGFR, and modulate downstream signaling pathways contributing to glioma progression.^[^ ^21^ ^]^ HBEGF is expressed by malignant glioma^[^ ^21^ ^]^ and has a role in gliomagenesis,^[^ ^22^ ^]^ drug resistance,^[^ ^23^ ^]^ and metastasis.^[^ ^24^ ^]^ HBEGF stimulates tumor growth and increases tumor blood vessel density and size.^[^ ^25^ ^]^ Chemotherapy upregulates tumor HBEGF expression, while silencing of HBEGF induces tumor cell apoptosis.^[^ ^26^ ^]^ HBEGF targeting showed promising results in Phase I clinical trials for solid tumors^[^ ^27^ ^]^ and EGFR, a central oncoprotein^[^ ^24^ ^]^ is clinically evaluated for glioma in temozolomide‐combination therapy.^[^ ^28^ ^]^ Administration of heparin (low molecular weight heparin/LMWH) post Stupp protocol was investigated in GB.^[^ ^29^ ^]^ LMWH did not increase temozolomide toxicity, nor induce deep vein thrombosis (DVT) or bleeding. One‐year overall survival significantly improved from 41.2% in the control group to 84.6% for LMWH treated patients. However, longer two‐year survival only showed a trend toward improvement in LMWH‐treated GB patients. Although these clinical trials show promise in GB, better longer‐term survival could be warranted by increasing HP delivery to the brain, overcoming BBB selectivity. Supporting this, an independent study recently demonstrated that nanobodies targeting HBEGF were successfully transported across a humanized in vitro BBB model.^[^ ^30^ ^]^

We engineered HP nanoparticles (HP‐NPs) as precision medicine targeting HBEGF with cargo delivering abilities across the BBB. We previously demonstrated that heparin‐derived nanocarriers efficiently cross physiological and pathological BBB, and specifically home to GB in preclinical models.^[^ ^31^ ^]^ HP‐derived nanocarriers were stable in blood and did not bind to blood plasma proteins or red blood cells after intravenous infusion in healthy rats.^[^ ^31^ ^]^ We adopted this study as a proof‐of‐concept to engineer nanoparticles to deliver doxorubicin (DOX) to GB. DOX was selected as a model drug following studies showing increased GB patient survival after intratumoral administration,^[^ ^32^ ^]^ since systemic infusion of DOX is unable to reach GB.^[^ ^33^ ^]^ In this study, we identified HP's capability to target MES‐like GB cells expressing HBEGF, reprogramming them into more vulnerable states and compromising tumor growth in preclinical GB patient avatars. The HP‐NPs we developed meet the essential criteria for clinical nanomedicine, specifically targeting glioma stem cells and influencing multiple pathways involved in GB progression and metastasis. HP‐NPs potential as a standalone treatment or in combination with existing therapies for brain cancer brings a novel approach to combat this aggressive disease.

## 2 Results

### 2.1 Synthesis and Characterization of HP‐NPs and Its Drug Release Profiles

**Figure 1:**
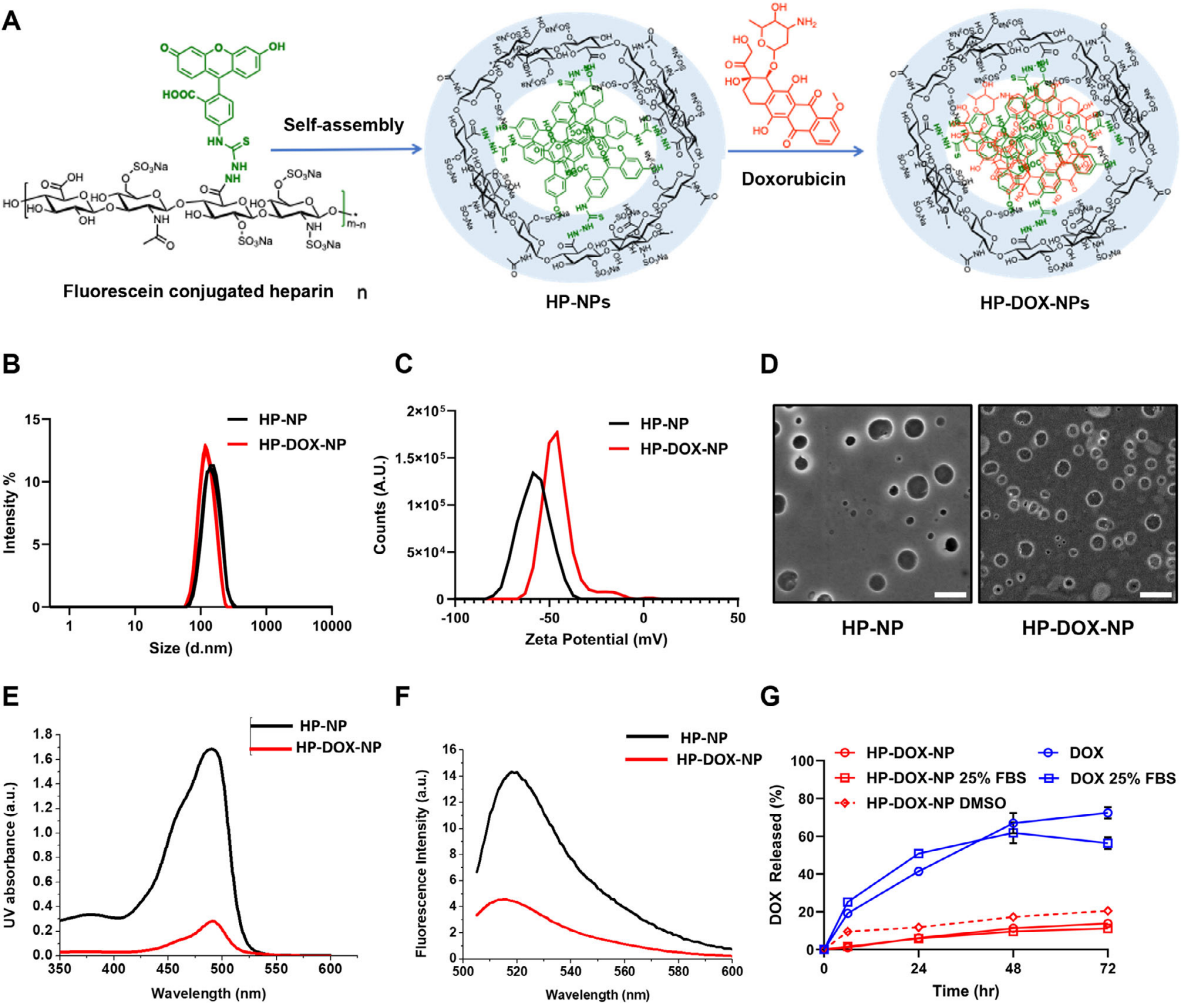
Heparin Nanoparticle synthesis and characterization. A) Graphical illustration of heparin nanoparticle self‐assembly and doxorubicin loading. B) DLS size distribution of HP‐NP and HP‐DOX‐NP at 1 mg mL^−1^ in 1x PBS (pH 7.4). C) Zeta potential distribution of HP‐NP and HP‐DOX‐NP at 0.1 mg mL^−1^ in 1x PBS (pH 7.4). D) SEM micrograph of gold sputtered self‐assembled HP‐NP and HP‐DOX‐NPs, scale = 500 nm. E) UV–vis absorbance spectra of HP‐NP and HP‐DOX‐NP at 1 mg mL^−1^ in diH_2_O. F) Fluorescence emission spectra of HP‐NP and HP‐DOX‐NP at 1 mg mL^−1^ in diH_2_O. G) DOX release kinetics of DOX and HP‐DOX‐NP in 1x PBS (pH 7.4) and 25% FBS in PBS, data represented by mean ± SD with *n* = 3 replicates.

We synthesized HP‐NPs by conjugating fluorescein to the HP polysaccharide backbone (**Figure** **1**A) following the reported protocol.^[^ ^34^ ^]^ Degree of fluorescein conjugation was tuned to form self‐assembled micelles with 150 nm hydrodynamic size in water. Degree of fluorescein modification was estimated at 1.8% with respect to disaccharide units of HP as quantified by UV–vis spectroscopy. Lyophilized, self‐assembled HP‐NPs, resuspended in 1x phosphate buffered saline (PBS), gave a hydrodynamic size of 146.9 ± 93.2 nm with a polydispersity index (PDI) value of 0.248 and –53.1 ± 7.39 mV zeta potential (Figure 1B,C). The anionic charge from HP's sulfate and carboxylate groups on the nanoparticle surface rendered excellent stability even upon lyophilization. Scanning electron microscopy (SEM) images of HP‐NPs and HP‐DOX‐NPs displayed particle structures ranging between 100–200 nm (Figure 1D). DOX was encapsulated within the hydrophobic core (Figure 1A) following nanoprecipitation method as reported earlier.^[^ ^34^ ^]^ Loading of DOX within the aromatic fluorescein core compacted nanoparticles to 117.1 ± 63.7 nm with a PDI of 0.215 and increased zeta potential to −48.7 ± 7.69 mV when resuspended in PBS. Drug loading in HP‐DOX‐NPs was estimated at 4.6% w/w using UV–vis as previously established.^[^ ^34^ ^]^ Strong *π*–*π* interaction between fluorescein core and DOX was evidenced from decreased UV and fluorescence signals (Figure 1E,F). Estimation of drug‐release kinetics in PBS (pH 7.4) and in PBS with 25% fetal bovine serum (FBS) displayed near zero‐order release kinetics of DOX from HP‐DOX‐NP for 72 h illustrating high stability in the presence of serum proteins, while free DOX demonstrated fast release from the dialysis membrane (Figure 1G). Addition of DMSO to HP‐DOX‐NPs accelerated the drug release. Drug loaded HP‐DOX‐NPs were stable and could be stored lyophilized for over six months at −20 °C without compromising stability and could be resuspended in saline before use.

### 2.2 Assessment of Anticoagulant Activity of HP‐NPs by Anti‐Factor Xa Assay

**Figure 2:**
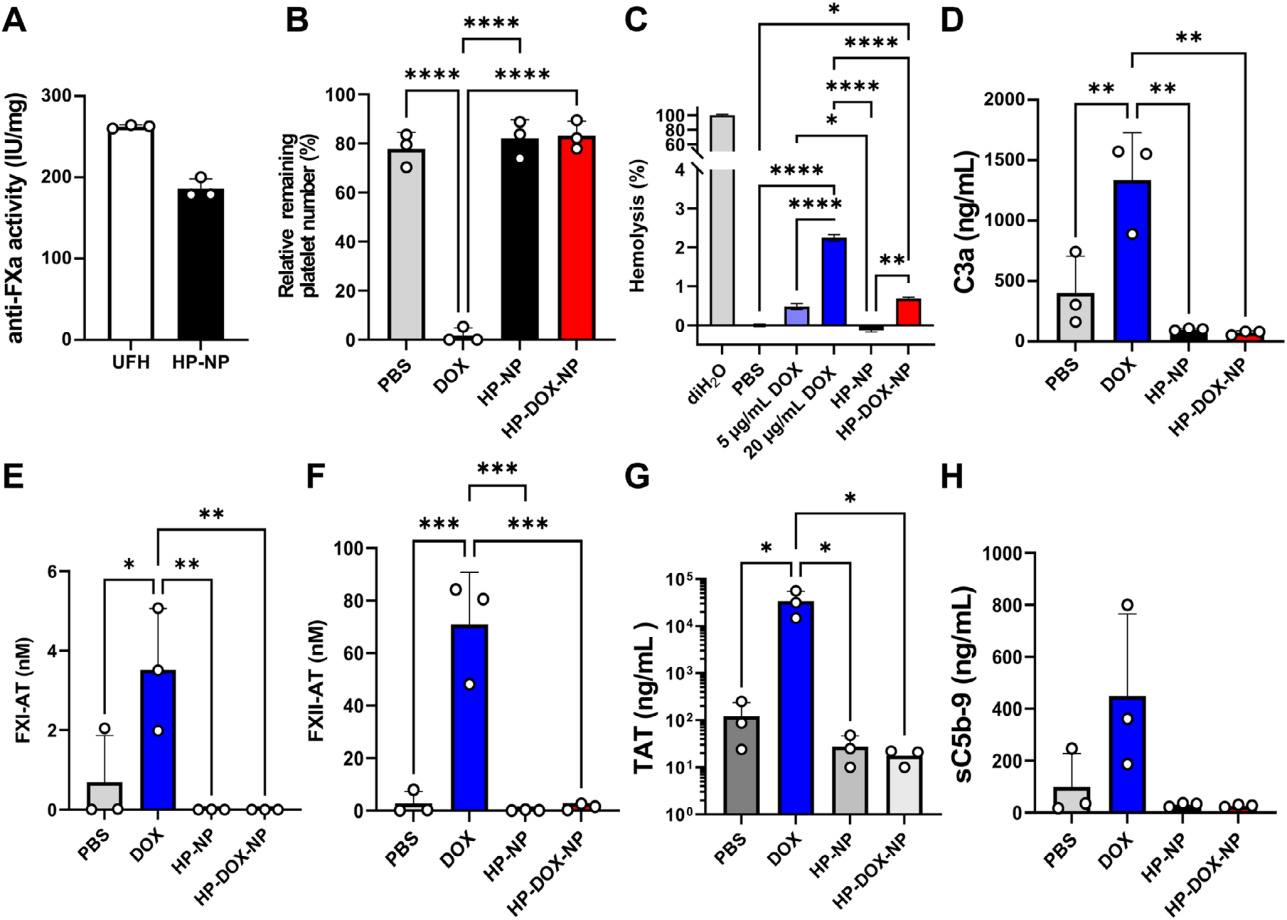
Hemocompatibility evaluation of HP‐NP and HP‐DOX‐NP in non‐coagulated human whole blood. A) Anti‐Factor Xa assay measuring anticoagulation properties. B) Human platelet toxicity assay using whole blood loop model following 60 min incubation at 37 °C. C) Hemolysis assay in human donor blood from *nn* = 3 patients with 3 technical replicates from each patient sample. D–H) ELISA quantification of complement cascade activation pathways in human whole blood, D. C3a activity (ng mL^−1^), E) FXI‐AT levels (nm), F) FXII‐AT (nm), G) TAT levels (ng mL^−1^), and H) sC5b‐9 levels (ng mL^−1^). For all other panels in Figure 2, human non‐coagulated whole blood was used from nn = 6 healthy human donors. Data presented as mean ± SD and analyzed with One‐Way ANOVA and Tukey's multiple comparisons test.

Anticoagulant properties of heparin induce bleeding. For effective use of HP‐NPs in cancer therapy, bleeding must be addressed. To ascertain the anticoagulant activity of HP‐NPs relative to unfractionated heparin (UFH), we estimated anti‐factor Xa activity in non‐anticoagulated human whole blood, obtained from six healthy donors. We utilized ex‐vivo human whole blood Chandler's loop model for this study following the reported protocol.^[^ ^35^, ^36^ ^]^ Compared to UFH, HP‐NPs displayed ≈30% reduction in anticoagulant activity. This was estimated by quantifying Factor Xa activity in human whole blood and found to be 186 ± 10 and 263 ± 1 [IU mg^−1^] after 1 h incubation (**Figure** **2**A). Thus, 1.8% modification of heparin backbone followed by core‐shell self‐assembly significantly reduces bleeding risk in patients.

### 2.3 Human Blood Hemocompatibility and Hemostability Assay of HP‐NPs and HP‐DOX‐NPs

We evaluated hemocompatibility and stability of DOX, HP‐NPs, HP‐DOX‐NPs in non‐anticoagulated human whole blood by measuring platelet count (Figure 2B), hemolysis % of red blood cells (RBCs) (Figure 2C), C3a (Figure 2D), FXI (Figure 2E), FXII (Figure 2F), TAT (Figure 2G), and sC5b‐9 (Figure 2H) with blood loop model following reported protocol.^[^ ^35^ ^]^ For platelet count assessment, we used 60 µm DOX or DOX equivalent in HP‐DOX‐NPs and compared it with equivalent amount of HP‐NPs, native UFH, and PBS as control. A marked drop in platelet count and macroscopic clotting was observed in DOX samples when incubated for 60 min at 37 °C. No reduction of platelets was observed in HP‐NP and HP‐DOX‐NP groups (Figure 2B).

We next assessed hemostability with a hemolysis assay using 0.1 mg mL^−1^ HP‐NPs and HP‐DOX‐NPs in fresh human donor blood along with two different doses of DOX at 5 µg mL^−1^ to match DOX loading in 0.1 mg mL^−1^ HP‐DOX‐NP, and higher dose of 20 µg mL^−1^ DOX using deionized H_2_O as positive control and PBS vehicle as negative control to establish lysis range following established protocol (Figure 2C).^[^ ^37^ ^]^ HP‐NP showed no hemolysis of RBCs returning lower absorbance values than negative PBS vehicle control. HP‐DOX‐NP showed slight hemolysis of 0.56% matching the lower 5 µg mL^−1^ DOX dose, which is well within accepted in vitro hemolysis range for drugs with low hemolytic risk at <10%.^[^ ^38^ ^]^ However, incubation of erythrocytes with 20 µg mL^−1^ DOX induced ≈2.5% hemolysis indicating toxicity of DOX at higher concentration. Thus, our hemolysis results illustrate high hemostability of HP‐NPs and HP‐DOX‐NPs with minimal to no hemolysis well within acceptable ranges in human donor blood.

We quantitated pro‐inflammatory response of DOX and HP‐DOX‐NP by measuring early and late markers of coagulation activation cascade, namely FXI‐antithrombin complex, FXII‐antithrombin complex, and thrombin‐antithrombin complex (TAT) (Figure 2E–G). We found DOX at 60 µm concentration significantly triggered coagulation cascade, as we observed elevated levels of FXI‐AT, FXII‐AT, and TAT. However, HP‐DOX‐NPs markedly attenuated this effect (Figure 2E–G). These results confirmed DOX‐induced acute platelet aggregation and coagulation activation. This was suggested to induce thrombocytopenia in DOX‐treated patients^[^ ^39^ ^]^ and can be prevented using our NP formulation strategy. We also found that DOX significantly triggered complement cascade with elevated levels of C3a and sC5b‐9 (Figure 2D,H). Complement activation, however, was completely mitigated upon nanoformulation. Our results suggest that encapsulation of DOX within HP‐NPs mitigate DOX‐mediated toxicity to human platelets and suppressed thromboinflammation and immune activation,^[^ ^32^, ^40^ ^]^ thus enhancing safety of chemotherapy.

### 2.4 In Vitro Evaluation of HP‐NPs Uptake in Patient Derived GB Stem Cells

**Figure 3:**
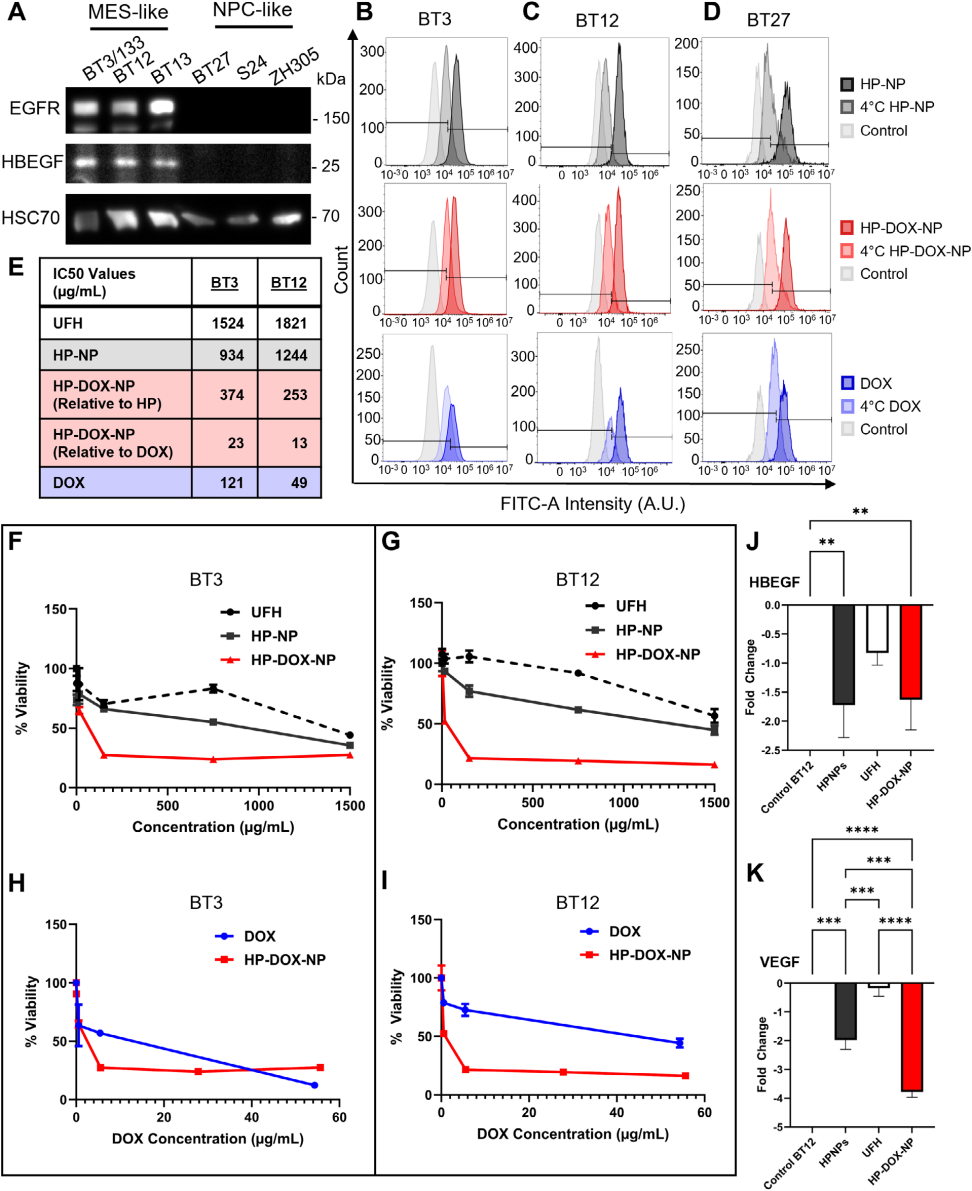
In vitro assessment of HP‐NP activity on human patient derived glioblastoma cells. A) Western Blot of EGFR, HBEGF, and heat shock chaperone protein 70 (HSC70) protein expression patient derived BT cell lines BT3/133, BT12, BT13 (MES‐like GSCs) and BT27, S24, ZH305 (NPC‐like GSCs). B–D) Quantification of HP‐NP, HP‐DOX‐NP, and DOX uptake following 2 h incubation using FACS from 10000 events on A. BT3 cells, B.  BT27 cells, C. BT12 cells. E,F) Cell viability of BT3 and BT12 cells following 48 h of HP‐NP exposure and incubation at 37 °C measured with MTT assay (*N* = 3). G,H) MTT cell viability of BT3 and BT12 cells following free DOX and HP‐DOX‐NP exposure (n = 3). I) Estimated in vitro IC50 values of BT3 and BT12 cells from cell viability assays (E‐F). J,K) Relative RT‐PCR analysis of HBEGF (J.) and VEGF (K.) expression in BT12 cells following 24 h exposure, to different HP‐treatments and compared to the untreated cells (n = 3). All data are mean ± SD, compared using One‐Way ANOVA with Tukey's multiple comparisons test in J. and K.

After establishing stability and safety of HP‐NPs, we tested and quantified HP‐NPs uptake in patient derived GSCs that express varying levels of EGFR in vitro.^[^ ^41^ ^]^ To confirm previously published total RNA sequencing data (Figure S1, Supporting Information), we conducted protein level analyses on cell extracts from MES‐ (BT3^CD133+^, BT12 and BT13) and NPC‐like (BT27, ZH305, and S24) GSCs (**Figure** **3**A). In accordance with the gene expression data (Figure S1, Supporting Information), all MES‐like cell lines exhibited high protein levels for EGFR and HBEGF, while these two markers were below detectable levels in NPC‐like cell lines (Figure 3A). We next selected BT3, BT12, and BT27 to challenge the hypothesis that HP‐NPs binding to HBEGF would suppress the HBEGF‐EGFR axis, a known driver of GB proliferation, resistance to apoptosis, and metastasis.^[^ ^42^, ^43^ ^]^

We evaluated HP‐NP, HP‐DOX‐NP, and DOX uptake in single live cells under different experimental conditions to decipher possible mechanisms of HP‐NP uptake using fluorescence‐activated cell sorting (FACS) measuring DAPI in PB450 channel followed by FITC positivity of single live GB cells (Figure 3B–D; Figures S2 and S3, Table S1, Supporting Information). GB cells were exposed to HP‐NPs and HP‐DOX‐NPs dissolved and dispersed in cell culture media at 0.1 mg mL^−1^ concentration and incubated for 2 h under physiological conditions at 37 °C and 4 °C. All GB cell lines displayed high cellular uptake of HP‐NPs (≥88.9% FITC positivity) and HP‐DOX‐NPs (≥91.3% FITC positivity) following 2‐h incubation at 37 °C under physiological conditions (Figure 3B–D; Figures S2 and S3, Table S1, Supporting Information). Incubation at 4 °C reduced uptake by ≥62% of HP‐NPs (≤34.8% FITC positivity) and HP‐DOX‐NPs (≤34.8% FITC positivity in all GB cells). This strongly suggested energy dependent endocytosis of HP‐NPs and HP‐DOX‐NPs by GB cells.

Next, GB cells were exposed to DOX dissolved and dispersed in cell culture media at 5 µg mL^−1^ concentration (matching DOX loaded onto 0.1 mg mL^−1^ HP‐DOX‐NP) and incubated for 2 h under physiological conditions at 37 and 4 °C. DOX uptake was quantified in live single cells using the same protocol as HP‐NP using FACS with PB450 DAPI viability separation followed by FITC positivity gating. DOX exposure lowered cell viability in BT3 (3144 events counted), BT12 (3685 events counted), and BT27 GB cells (3733 counts) after live gating compared to control groups (6421 counts for BT3 control, 6756 counts in BT12 control, and 5387 for BT27) (Table S1, Supporting Information). All remaining living GB cells showed high DOX uptake with BT3 cells with the lowest DOX uptake (73.5% FITC positivity), followed by BT27 (97.5% FITC positivity), and BT12 (99.4% FITC positivity) following 2 h incubation at 37 °C. Moreover, exposure to DOX at 4 °C for 2 h reduced DOX uptake ≈50% across all GB lines. Exposure to DOX at 4 °C for 2 h resulted in an ≈50% reduction in DOX uptake across all GB cell lines tested (BT3: 4359 counts, BT12: 3662 counts, and BT27: 6362 counts). This finding aligns with previous literature indicating that lower temperatures can significantly impair cellular uptake of DOX. Specifically, Steenhoven et al. demonstrated that cold temperature not only reduces DOX internalization but also enhances cell survival, suggesting a dual effect of temperature on drug efficacy and cytotoxicity. The reduced uptake observed in our study is likely due to temperature‐dependent inhibition of active transport mechanisms and changes to membrane fluidity and rigidity, which are critical for DOX internalization. Additionally, the diminished cellular damage at 4 °C further supports the notion that cold exposure mitigates DOX‐induced cytotoxicity, potentially by limiting drug accumulation within the cells.^[^ ^44^ ^]^

To determine in vitro cell cytotoxicity of UFH, HP‐NP, HP‐DOX‐NPs, and DOX on MES‐like GB cells overexpressing EGFR (BT3 and BT12),^[^ ^33^ ^]^ we estimated the IC50 values using MTT cell viability assay after 48 h incubation (Figure 3E). Figure 3F,G illustrates dose response curves of UFH, HP‐NP, and HP‐DOX‐NP relative to the polymer/nanoparticle concentration exposure on GSCs from which IC50 values were estimated using non‐linear regression curve fitting and reported in Figure 3E. Figure 3H,I depicts dose response curves of DOX and HP‐DOX‐NP relative to DOX concentration. Thus, the HP‐DOX‐NP IC50 values tabulated in Figure 3E display two IC50 values where the first refer to cytotoxicity relative to the nanoparticle concentrations (panels 3F and 3G) and the second IC50 values refer to cytotoxicity relative to the DOX concentration (panels 3H and 3I). We observed that HP‐NPs increased cytotoxicity in GB cells as compared to UFH, although to a lower extent than HP‐DOX‐NPs or DOX. In BT3 cells, IC50 value for UFH was 1524 µg mL^−1^ while HP‐NPs reached IC50 toxicity at 934 µg mL^−1^. HP‐NP exposure resulted in higher toxicity compared to UFH also in BT12 cells with IC50 values of 1821 and 1244 µg mL^−1^ for UFH and HP‐NPs, respectively. HP‐DOX‐NPs displayed higher potency than DOX as evident from increased cytotoxicity at lower required doses (Figure 3E) as shown in dose response curve (Figure 3H,I). In BT3 cells, 374 µg mL^−1^ of HP‐DOX‐NP with 23 µg mL^−1^ of loaded DOX concentration exposure was needed to reach IC50 compared to DOX with an estimated IC50 value at 121 µg mL^−1^. In BT12 cells, the toxicity of HP‐DOX‐NP was more pronounced with an IC50 value of 253 µg mL^−1^ HP‐DOX‐NP concentration with 13 µg mL^−1^ of loaded DOX present. DOX IC50 value in BT12 cells was estimated at 49 µg mL^−1^. DOX loading into HP‐NPs led to around five‐fold reduction in required DOX dosage to reach IC50 in BT3 cells and around four‐fold reduction in DOX dose in BT12 cells to reach same potency as free DOX. DOX encapsulation into HP‐DOX‐NPs increased cytotoxic effect of DOX with lower doses of DOX required.

HP is a known anti‐angiogenic downregulating VEGF expression.^[^ ^47^, ^48^ ^]^ HP is a natural ligand for HBEGF, which contributes to tumor angiogenesis and regulates tumor vessel density.^[^ ^25^ ^]^ We hypothesized HP‐NPs would modulate proangiogenic signals produced by GB cells, including VEGF and HBEGF. Based on total RNA sequencing gene expression analyses, BT12 among all available GSCs appeared to upregulate most genes related to VEGFRs signaling (Figure S1, Supporting Information). Using RT‐qPCR, we evaluated expression of VEGF and HBEGF in BT12 cells following incubation with HP‐NPs, UFH, and HP‐DOX‐NPs for 24 h as compared to untreated control cells (Figure 3J,K). HP‐NPs and HP‐DOX‐NPs significantly downregulated HBEGF (around two‐fold) compared to untreated control BT12 cells. UFH led to a nonsignificant 0.8‐fold reduction in HBEGF expression. VEGF expression was downregulated upon HP‐NPs (two‐fold) and HP‐DOX‐NP (four‐fold) exposure compared to untreated control or UFH. Nanoformulation of HP led to greater downregulation of oncogenic pathways in GB cells in vitro as compared to UFH alone, justifying preclinical evaluation in laboratory animals.

### 2.5 Safety Assessment of HP‐NPs in Healthy Male and Female Rats

To evaluate HP‐NPs' pharmacokinetics and safety, we administered high (20 mg kg^−1^) or low (2 mg kg^−1^) doses of HP‐NPs to adult male and female Wistar rats daily for 14 days. We adopted this intensive treatment regime to estimate the maximum tolerability/toxicity by daily injection of HP‐NPs. During the study, rats showed no weight loss, instead showed increase in body weight (Table S2, Supporting Information). However, we observed increased bleeding in high‐dose rats which was considerably reduced in the low dose group (3/10 rats).

**Figure 4:**
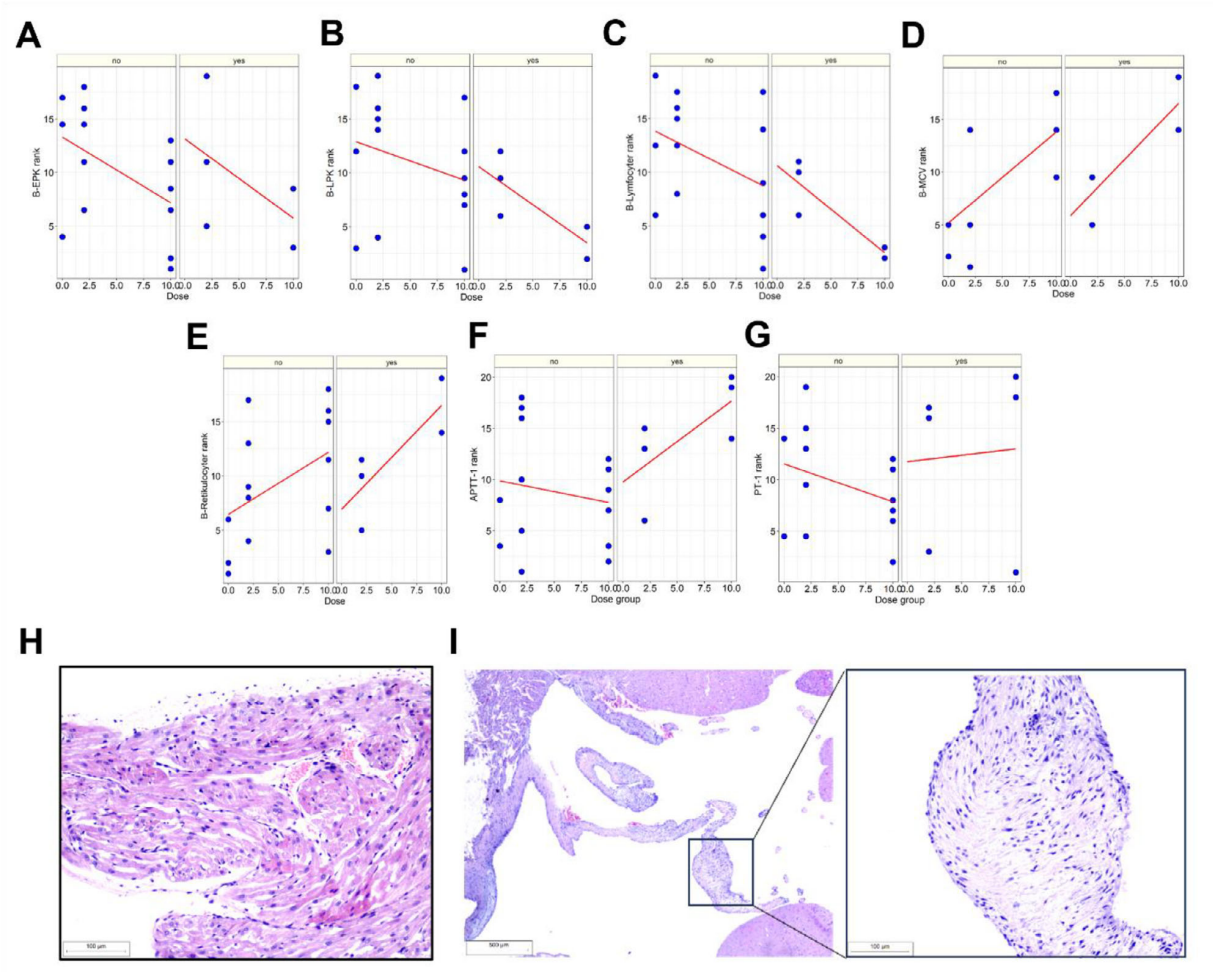
Preclinical Safety Assessment of HP‐NP injections in rats. A) Erythrocyte particle concentration (EPK) after 14 days treatment with HP‐NP was affected by dose (*p* = 0.024) and by sex (*p* = 0.0107), but not whether a blood sample was collected during the study (*p* = 0.92). B) Leucoyte particle concentration (LPK) after 14 days treatment with HP‐NP was affected by dose (*p* = 0.034) and by sex (*p* = 0.001), but not whether a blood sample was collected during the study (*p* = 0.61). C) Lymphocyte particle concentration after 14 days treatment with HP‐NP was affected by dose (*p* = 0.0057) and by sex (*p* = 0.0006), but not whether a blood sample was collected during the study (*p* = 0.37). D) Mean corpuscular volume (MCV) after 14 days treatment with HP‐NP was affected by dose (*p* = 0.0029) but not by sex (*p* = 0.52) or whether a blood sample was collected during the study (*p* = 0.82). E) Reticulocyte count after 14 days treatment with HP‐NP was affected by dose (*p* = 0.03) but not whether a blood sample was collected during the study (*p* = 0.84), or by sex (*p* = 0.083). For A‐E: Linear regression with 2‐way ANOVA approach, with Dose as the continuous factor, blood sample as the categorical factor and sex as a blocking factor. The response was transformed to rank prior to analysis. HP‐NP 2 mg kg^−1^ (*n* = 8), 10 mg kg^−1^ (*n* = 8), placebo (*n* = 3). F) The effect of HP‐NP on activated partial prothrombin time (APTT) after daily treatment for 14 days in rats. Neither treatment nor blood sampling, nor sex had an effect on APTT (*p* = 0.37, 0.98 and 0.74), linear regression with 2‐way ANOVA approach (rank transformed response), with dose as the continuous factor, blood sample as the categorical factor and sex as a blocking factor. HP‐NP 2 mg kg^−1^ (*n* = 9), 10 mg kg^−1^ (*n* = 9), placebo (*n* = 2). G) The effect of HP‐NP on partial thromboplastin time (PT) after daily treatment for 14 days in rats. Neither treatment nor blood sampling influenced PT (*p* = 0.99 and 0.92), whereas sex had (*p* = 0.02), linear regression with 2‐way ANOVA approach (rank transformed response), with dose as the continuous factor, blood sample as the categorical factor and sex as a blocking factor). HP‐NP 2 mg kg^−1^ (*n* = 8), 10 mg kg^−1^ (*n* = 7), placebo (*n* = 2). For A‐G: yes = blood sample collected during study, no = no blood sample collected.  H) Left atrium of the heart in rat 7 (female) after 14 days of 2 mg kg^−1^ HP‐NP administration. Vacuolar myocyte degeneration graded with a score 2 (see supplement 2 for details). HE‐staining, 100x magnification. I) HE‐staining of the aortic valve in rat 14 (male) in dose group 10 mg kg^−1^, showing myxomatous degeneration with a grade 2 (see supplement 2 for details). Left side 20x, enlargement 100x magnification.

Careful analysis of blood parameters before termination of the experiments (day 15), indicated minor effects on erythrocyte, leukocyte, and lymphocyte counts that were negatively correlated to dose. The reticulocyte counts and the mean red cell corpuscular volume (MCV) increased with higher dose (**Figure** **4**A–G) and the response variables were correlated (correlation coefficient 0.7, *p* < 0.001, Pearson's product moment correlation coefficient). One high dose female showed a lower white cell count than all other rats. This rat also had a lower number of lymphocytes, hemoglobin, and erythrocytes, and showed increased bleeding on day 5. Heparin inhibits factors X and thrombin, while activating anti‐thrombin and increases APTT dose dependently for ≈60 min in rats.^[^ ^49^ ^]^ In our safety study, hemoglobin, hematocrit and platelet counts were unaffected on the final day (day 15) suggesting no adverse thrombotic complication of HP therapy.

Clinical chemistry indicated liver and kidney toxicity in this female high‐dose rat (elevated ALAT, ASAT, creatinine, urea), while others were unaffected. Histopathology revealed mild cardiotoxicity, including cardiomyocyte vacuolation and myxomatous valve changes, more pronounced in high‐dose rats. No inflammation or organ lesions in liver, kidney, lungs, or spleen were observed. For complete hematology and clinical chemistry see Tables S3–S7 (Supporting Information) for individual values from each animal.

Next, we performed gross pathological evaluation of the high dose and low dose groups, however, it did not reveal any significant findings. Histopathology showed mild cardiotoxicity in form of cardiomyocyte cytoplasmic vacuolation (Figure 4H,I) that was more pronounced in the high dose group (no statistical evaluation was performed). However, no signs of inflammation, edema, fibroplasia, or fibrosis were observed. Further analysis of cardiotoxicity indicated myxomatous change in both the left atrioventricular valve and the aortic valve (Figure 4H,I). The score was higher in the high dose group than the low dose group. This could be a direct toxic effect, or caused by hemorrhage, which increases heart rate and is known to induce dominantly left‐sided myxomatous valvular lesions. There were no lesions in kidney, liver, lung or spleen that would be indicative of any tissue toxicity. We anticipate the intensity of toxicity would be reduced when three‐day interval is applied between each administration (mimicking our treatment regime), which will facilitate recovery and establish hemostasis.

### 2.6 Preclinical Evaluation of HP‐NPs in Human GB Patient Avatars

**Figure 5:**
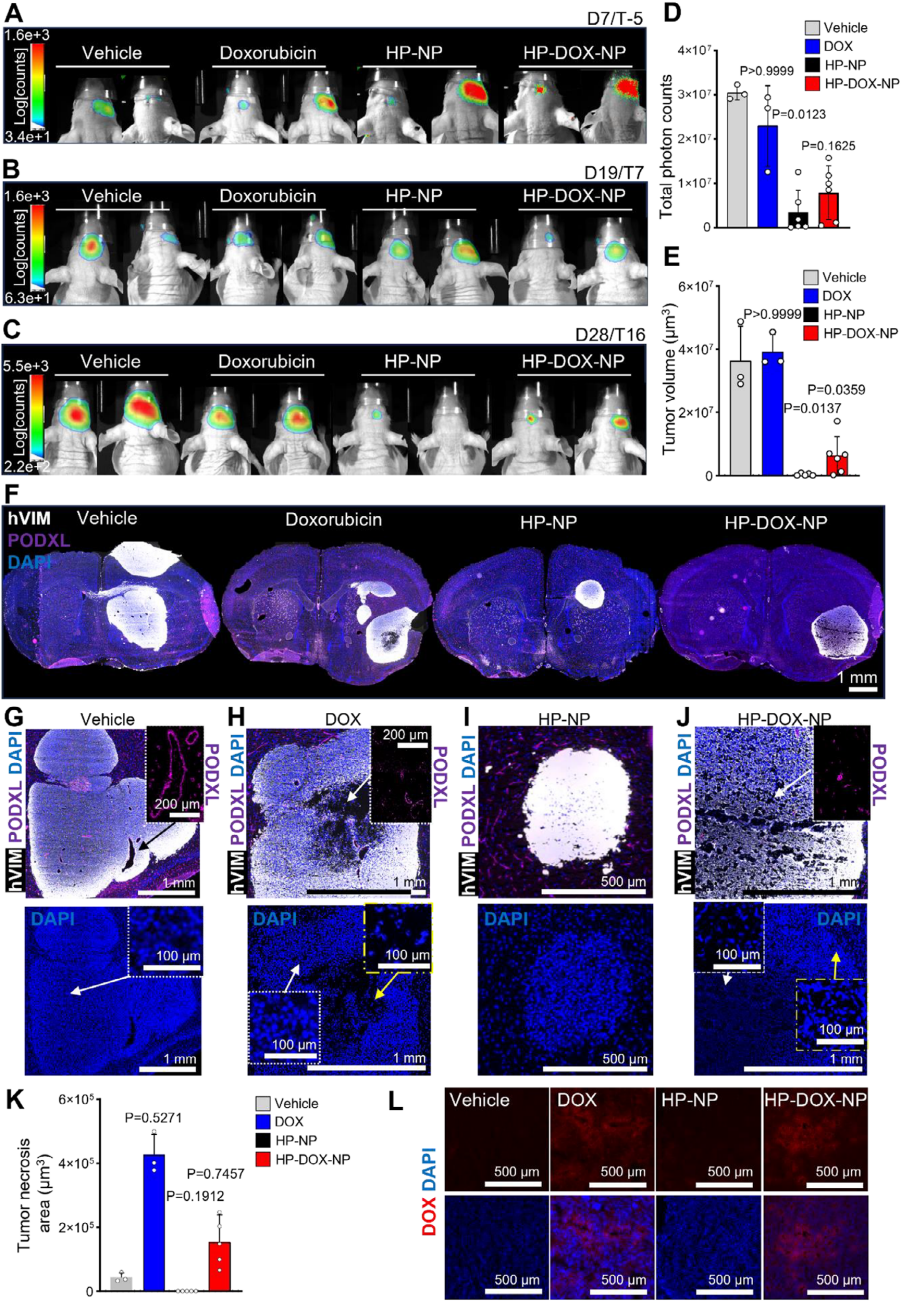
Preclinical characterization of HP‐NPs in IDH WT glioblastoma patient avatars. A) representative live bioluminescence imaging of BT12 patient derived GB stem cells expressing luciferase, implanted in the mouse brain striatum. Imaging performed seven days post implantation (D7) and five days prior to the treatment initiation (T‐5). B) representative live bioluminescence imaging of BT12 patient derived GB stem cells expressing luciferase, implanted in the mouse brain striatum. Imaging performed nineteen days post‐implantation (D19) after three rounds of caudal vein infusions (T7) with vehicle saline (n = 3, 200 µL), doxorubicin (n = 3, 200 µL, 1.5 mg kg^−1^), heparin nanoparticles (HP‐NP, n = 6, 200 µL, 5 mg kg^−1^) or doxorubicin‐loaded heparin nanoparticles (HP‐DOX‐NP, n = 6, 200 µL, 5 mg kg^−1^). C) representative live bioluminescence imaging of BT12 patient derived GB stem cells expressing luciferase, implanted in the mouse brain striatum. Imaging performed after twenty‐eight days (D28) and six rounds of caudal vein infusions (T16) with vehicle saline (n = 3, 200 µL), doxorubicin (n = 3, 200 µL, 1.5 mg kg^−1^), heparin nanoparticles (HP‐NP, n = 6, 200 µL, 5 mg kg^−1^) or doxorubicin‐loaded heparin nanoparticles (HP‐DOX‐NP, n = 6, 200 µL, 5 mg kg^−1^). D) quantification of the intravital photon counts on day 28. Vehicle patient avatars (n = 3), doxorubicin (DOX) 1.5 mg kg^−1^ (n = 3), HP‐NPs (5 mg kg^−1^, n = 6) and HP‐DOX‐NP (n = 6). E) tumor volume determined from histological sections (ten per mouse) of mouse brain avatars treated with vehicle (n = 3), DOX (n = 3), HP‐NP (n = 5) and HP‐DOX‐NP (n = 6). F) representative immunofluorescence pathology micrographs of Vehicle, DOX, HP‐NP, and HP‐DOX‐NP treated patient avatars brain section labelled for human vimentin (hVIM, white), mouse endothelial podocalyxin (PODXL, magenta) and DAPI (blue). G) close‐up micrograph of the typical pathology for BT12 patient avatars, featuring enlarged tumor blood vessels (top right insert) devoid of necrotic features (bottom image) or nuclear atypia (arrow‐pointing insert). H) close‐up micrograph of the typical pathology for BT12 patient avatars treated with doxorubicin, featuring cell and blood‐vessel‐free intratumoral areas (top right insert) and presenting necrotic features (bottom image) with pyknotic‐like nuclei (arrow‐pointing yellow insert) when compared to non‐necrotic tumor areas (arrow‐pointing white insert). I) close‐up micrograph of the typical pathology for BT12 patient avatars treated with HP‐NP, featuring smaller tumors devoid of cellular atypia cell and blood‐vessel‐free intratumoral areas (top right insert) and presenting necrotic features (bottom image) with nuclear atypia (arrow‐pointing yellow insert) when compared to non‐necrotic tumor areas (arrow‐pointing white insert). J) close‐up micrograph of the typical pathology for BT12 patient avatars treated with HP‐DOX‐NP, featuring histological discontinuity associated with pyknotic nuclei (yellow insert) compared to unaffected tumor areas (white insert)and blood‐vessel‐free intratumoral areas (top right insert) and presenting necrotic features (bottom image) with nuclear atypia (arrow‐pointing yellow insert) when compared to non‐necrotic tumor areas (arrow‐pointing white insert). K) quantification of necrotic features, data presented as necrotic area (µm^2^) averaged from two brain sections per vehicle, DOX, HP‐NP, and HP‐DOX‐NP treated patent avatars. Panels D, E, and K: statistical significance was determined by one‐way ANOVA with Kruskal‐Wallis post hoc test for multiple comparisons of treatment versus vehicle. L) Representative fluorescent micrographs for doxorubicin autofluorescence (470/595 nm) in Vehicle, DOX, HP‐NP, and HP‐DOX‐NP treated patient avatars brain section counterstained with DAPI.

We assessed anti‐tumor efficacy of HP‐NP and DOX loaded HP‐DOX‐NP in a clinically relevant model of GB, called patient avatar. Patient‐derived BT12 expressing luciferase were implanted in right brain striatum of immunocompromised NMRI/Nrj nude mice (**Figure** **5**).^[^ ^41^ ^]^ Twelve days post‐implantation, animals were infused (600 µL/min) three times per week in the caudal vein with either saline vehicle (200 µL), UFH (5 mg kg^−1^, 200 µL), DOX (1.5 mg kg^−1^, 200 µL), HP‐NP (5 mg kg^−1^, 200 µL) or HP‐DOX‐NP (5 mg kg^−1^, 200 µL). Treatment regimen was maintained for two weeks. Tumor progression was monitored by intravital bioluminescence imaging of avatars following intraperitoneal injection of luciferin (Figure 5A–D). When ethical limit was reached (from day 28 onwards^[^ ^41^ ^]^), avatars were euthanized, cerebral tissue collected and snap‐frozen for histological analyses. Intravital imaging revealed moderate response to DOX and decrease of the tumor‐associated bioluminescence in HP‐NPs and HP‐DOX‐NP groups (Figure 5A–D). Histological analyses using human‐cell specific antibodies were consistent with previous data, showing vehicle‐treated BT12 avatars exhibited heterogenous progression featuring bulk‐like tumor masses and extensive invasion of cerebral parenchyma (Figure 5E,F).^[^ ^41^ ^]^ DOX‐treated mice did not exhibit a significant reduction of tumor mass (Figure 5D–F). Strikingly, xenografts from HP‐NP and HP‐DOX‐NP treated groups underwent significant reduction of tumor growth, accompanied with notable decrease of tumor bulk sizes (Figure 5D–F). In addition, all samples from the DOX and HP‐DOX‐NP groups featured extensive necrotic areas, visualized by extensive gaps in the tissue and pyknotic nuclear counterstain within tumor tissue (Figure 5G–K) likely responsible for decreased bioluminescence in these groups (Figure 5A–D). Histological inspection of DOX autofluorescence (excitation at 470 nm, emission at 595 nm) confirmed that both DOX and HP‐DOX‐NP very likely permeated through larger, structurally aberrant and potentially leaky blood vessels at the edge and within necrotic areas (Figure 5L).

### 2.7 In Vivo Tumor Cell Fates Modulation by HP‐NPs

**Figure 6:**
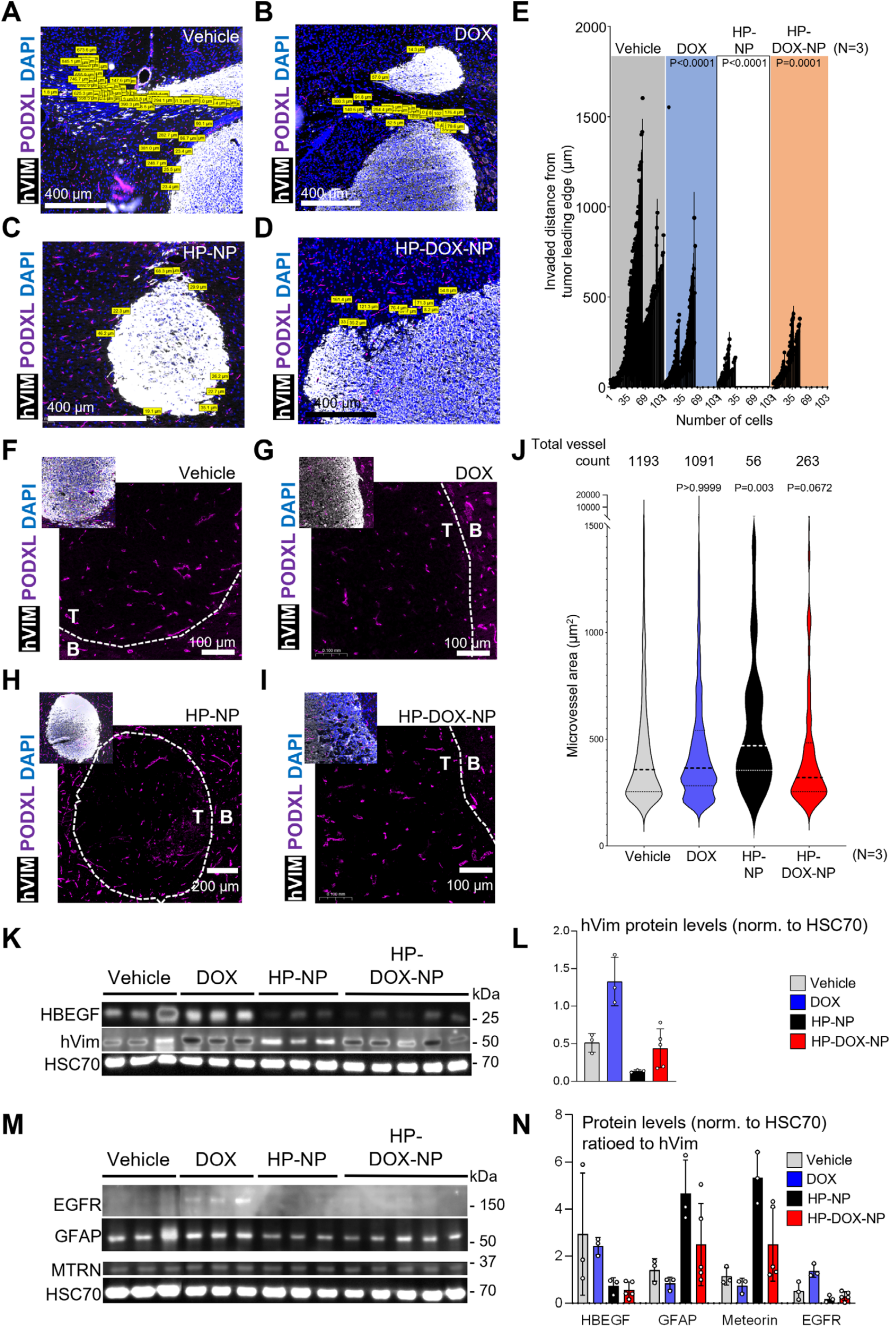
In vivo cellular fates and molecular dynamics coordinated by HP‐NPs treatments. A) representative micrograph of BT12 patient avatar cell invasion trajectories (hVIM and yellow boxes) along the *corpus callosum* of the host mouse brain (PODXL, mouse microvascular endothelium, magenta and DAPI, blue). B) representative micrograph of DOX‐treated BT12 patient avatar cell invasion trajectories (hVIM and yellow boxes) along the *corpus callosum* of the host mouse brain (PODXL, mouse microvascular endothelium, magenta and DAPI, blue). C) representative micrograph of HP‐NP‐treated BT12 patient avatar cell invasion trajectories (hVIM and yellow boxes) at the leading edge of the tumor. D) representative micrograph of HP‐DOX‐NP‐treated BT12 patient avatar cell invasion trajectories (hVIM and yellow boxes) along the *corpus callosum* of the host mouse brain (PODXL, mouse microvascular endothelium, magenta and DAPI, blue). E) quantification of the tumor cell dissemination (number of invasive cells, distance from the tumor leading edge) in vehicle, DOX, HP‐NP, or HP‐DOX‐NP treated BT12 patient avatars (*n* = 3 per group). F) representative micrograph of BT12 patient avatar microvascular endothelium architecture (hVIM, human tumor cells, white; PODXL, microvascular endothelium, magenta; DAPI, blue). T, intratumoral, B, healthy brain parenchyma. G) representative micrograph of DOX‐treated BT12 patient avatar microvascular endothelium architecture (hVIM, human tumor cells, white; PODXL, microvascular endothelium, magenta; DAPI, blue). T, intratumoral, B, healthy brain parenchyma. H) representative micrograph of HP‐NP‐treated BT12 patient avatar microvascular endothelium architecture (hVIM, human tumor cells, white; PODXL, microvascular endothelium, magenta; DAPI, blue). T, intratumoral, B, healthy brain parenchyma. I) representative micrograph of HP‐DOX‐NP‐treated BT12 patient avatar microvascular endothelium architecture (hVIM, human tumor cells, white; PODXL, microvascular endothelium, magenta; DAPI, blue). T, intratumoral, B, healthy brain parenchyma. J) quantification of the tumor microvascular endothelium (number of vessels per section, blood vessel area in µm^2^) in vehicle, DOX, HP‐NP, or HP‐DOX‐NP treated BT12 patient avatars (n = 3 per group). K) Western Blot of HBEGF, human vimentin (hVim) and heat shock chaperone protein 70 (HSC70) protein expression in vehicle, DOX, HP‐NP, and HP‐DOX‐NP treated patient avatars brain extracts. L, hVimentin protein levels ratioed to the HSC70 protein loading control. M) Western Blot of epidermal growth factor receptor (EGFR), glial fibrillary acidic protein (GFAP), meteorin (MTRN) and HSC70 protein expression in vehicle, DOX, HP‐NP and HP‐DOX‐NP treated patient avatars brain extracts. Image was edited between lanes three and four to mask the protein weight ladder lane. N) HBEGF, GFAP, MTRN, and EGFR protein levels normalized to the HSC70 protein loading control and ratioed to the human tumor cell abundance (e.g., hVim) in the brain extracts. Panels E, and J: statistical significance was determined by one‐way ANOVA with non‐parametric Kruskal‐Wallis post hoc test for multiple comparisons of treatment versus vehicle.

We next assessed the impact of the treatments on the intratumoral cellular and molecular dynamics. First, we dissected the intensity and distance of tumor invasion by quantitating disseminated human vimentin‐positive tumor cells in the mouse brain parenchyma (**Figure** **6**A–E). When compared to control tumors (Figure 6A,E), all treatment groups featured significantly reduced brain parenchyma dissemination (Figure 6B–E). To determine if the treatments also affected stromal cells such as the microvascular endothelium, we quantitated the number of podocalyxin‐positive vessels in the tumor bulks as well as their sizes (Figure 6F–J). When compared to control samples (Figure 6F,J), only the HP‐NP group featuring very small tumor bulk (Figure 6G–J) included slightly larger blood vessels on average (Figure 6J). However, only the vehicle and DOX‐treated groups exhibited extremely enlarged endothelial structures (>10 000 µm^2^, Figure 6J) as regularly observed across the histological sections (Figure 5G,H). Delving deeper in the tumor reorganization by HP‐NPs, we screened for key oncogenic protein markers in brain sample extracts. Protein levels for HBEGF, human tumor cell markers (vimentin), EGFR and astroglial differentiation markers (GFAP, Meteorin) were determined by Western Blot in all treatment groups (Figure 6K–N). We detected a decrease of human vimentin abundance (Figure 6K,L) consistent with the decrease in tumor size (Figure 5). DOX treated samples exhibited a trend for vimentin and HBEGF protein level increase (Figure 6K–N). Interestingly, HBEGF protein abundance appeared decreased in the HP‐NP and HP‐DOX‐NP groups, when ratio to human cell abundance using vimentin as reference (Figure 6K–N). We finally screened for additional classical/astroglial GB markers including the glial fibrillary acidic protein (GFAP), the glial differentiation marker meteorin and EGFR (Figure 6M). Interestingly, HP‐NP and HP‐DOX‐NP appeared to feature increased glial‐like features, suggested by increased protein levels for GFAP and meteorin compared to either vehicle of DOX groups, when normalized to human cell abundance (ratio to vimentin levels) (Figure 6M,N). A trend for EGFR upregulation was detected only in DOX‐treated brain extracts (Figure 6M,N). Consistent with the upregulation of vimentin (Figure 6L) and necrotic areas in the tissue samples (Figure 5), this pointed toward increased aggressiveness/chemoresistance with fortified mesenchymal features fueled by DOX treatments in vivo.

### 2.8 HP‐NP imposes mesenchymal to glial GSC phenotypic cell shift and decreased HBEGF/EGFR pathway gene expression levels

**Figure 7:**
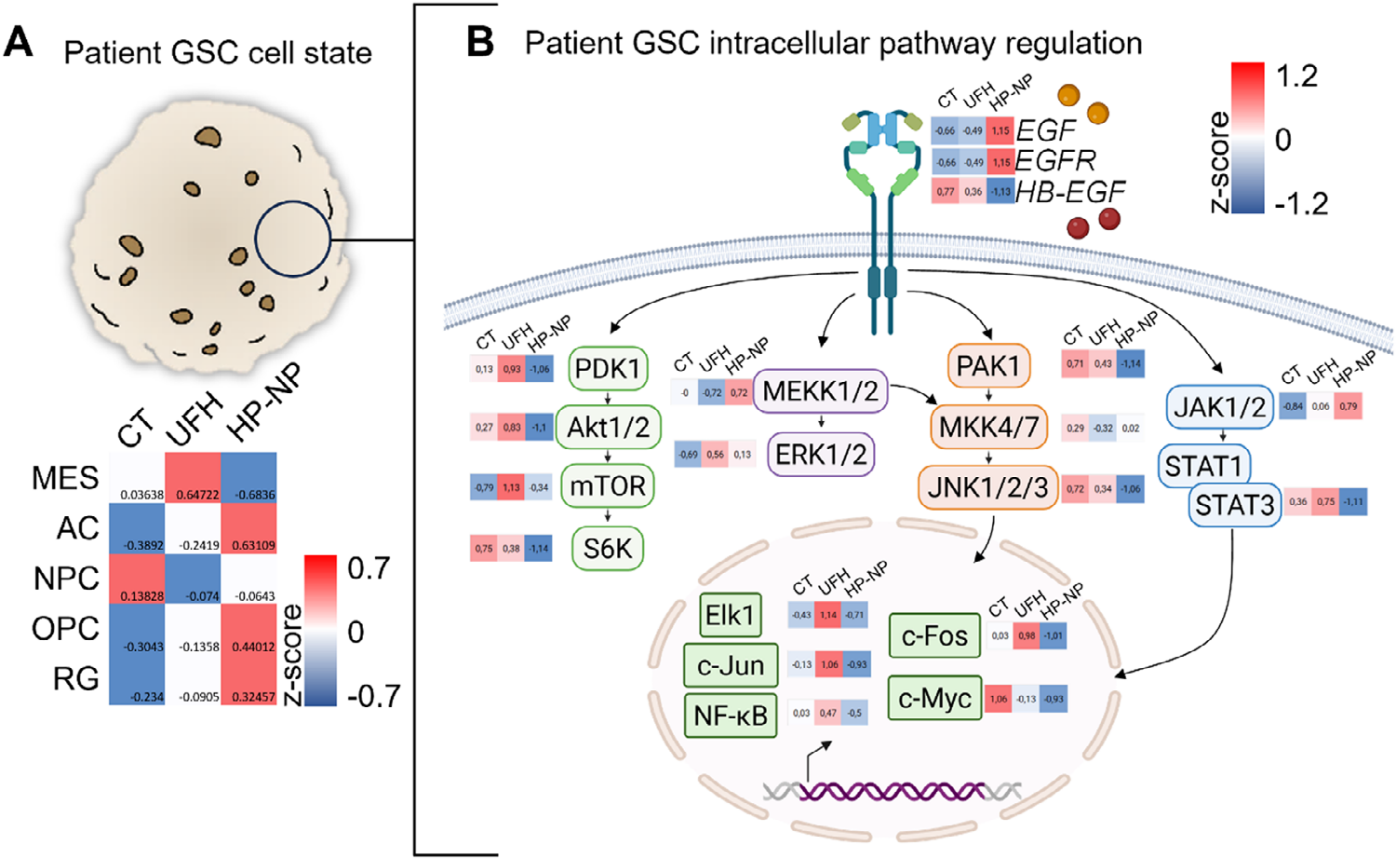
HP‐NPs induces glioma stem cell differentiation and phenotypic cell state shift from mesenchymal‐like to astrocyte‐like glioblastoma. A) Gene expression profiling of phenotypic cell states dynamics in control (CT), UFH or HP‐NP treated BT12 GSCs (24 h). Gene expression signature according to Neftel et al., 2019. Z‐scores calculated with normalized raw count values from total RNA sequencing. MES, mesenchymal‐like, AC, astrocyte‐like, NPC, neuron precursor‐like, OPC, oligodendrocyte precursor cell‐like, RG, radial glia‐like. B) Overview of the gene expression level for the HBEGF/EGFR signaling pathway in BT12 when grown for 24 h in control (CT) cell culture condition and in response to unfractionated heparin (UFH) and heparin nanoparticle (HP‐NP). Z‐scores calculated with normalized raw count values from total RNA sequencing.

An in‐depth characterization of GSC transcriptomic modifications induced by treatments were performed employing total RNA sequencing of BT12 cell samples cultured in control, UFH, HP‐NP, DOX, and HP‐DOX‐NP supplemented conditions for 24 to 48 h (**Figure** **7**; Figure S4, Supporting Information). Transcriptomic profiling of GSC phenotypic cell states as described by Neftel et al. in 2019,^[^ ^1^ ^]^ radial glia‐like gene expression and HBEGF expression was investigated. UFH, DOX, and HP‐DOX‐NP treatments appeared to fortify original mesenchymal‐like state of BT12 (Figure 7A; Figure S4, Supporting Information), while HP‐NP induced a marked phenotypic shift toward astroglial‐like cell differentiation, including astrocyte‐like, oligodendrocyte precursor cell like, and radial glia‐like cell states (Figure 7A) in accordance with the glial marker protein levels detected in the mouse brain extracts (Figure 6N,M). When further investigating the HBEGF/EGFR pathways gene expression regulation, we uncovered that *HBEGF* gene expression was specifically and markedly reduced by HP‐NP treatments (Figure 7B). This was accompanied with a global downregulation of the main intracellular pathways connected to EGFR activation (Figure 7B). This included decreased gene expression of the mTOR, AKT, and JAK/STAT pathways, well characterized for their contribution to GB aggressiveness and mesenchymal cellular states. Together, these data shed light on the ability of HP‐NP to orchestrate the loss of mesenchymal‐like stem cell traits in favor of more differentiated cell states.

While transcriptomic analyses included DOX and HP‐DOX‐NP, those groups were characterized with high cell toxicity (as described in Figure 3) generating lower quality samples for total RNA sequencing. This technical bias prevented the accurate characterization of the phenotypic cell state shifts (Figure S4, Supporting Information). However, the observed transcriptomic fortification of mesenchymal features detected in DOX and HP‐DOX‐NP treated cells compared to the HP‐NP group would be supported by the preclinical data in patient avatars (Figures 5, 6). To further understand the interplay between HBEGF expression and mesenchymal GBs, we expanded our investigations to include publicly available human databases.

### 2.9 HBEGF is a Strong Predictor of Mesenchymal Gliomas Associated with Reduced Survival in Patients

**Figure 8:**
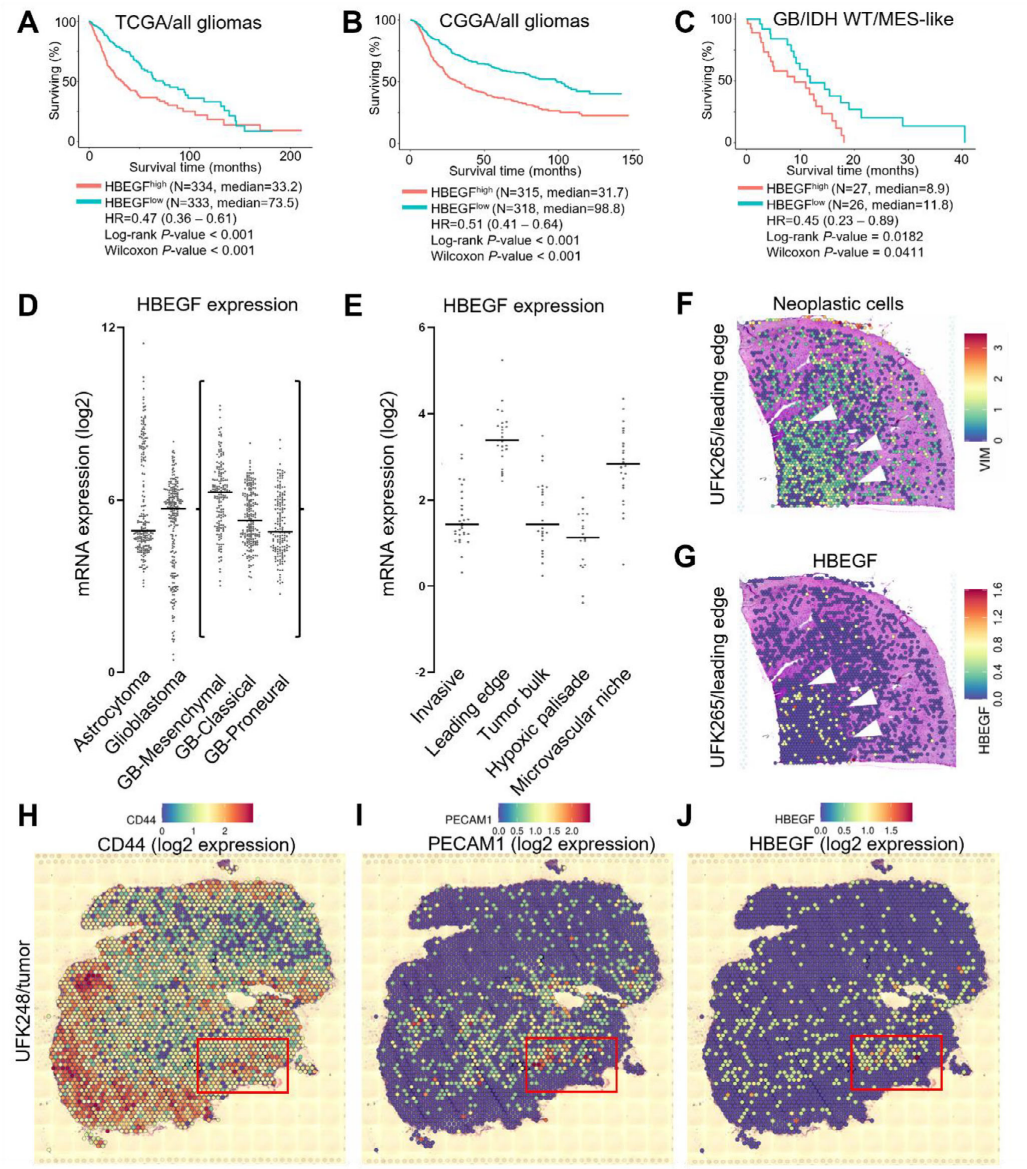
HBEGF is a patient biomarker of fast progressing mesenchymal‐like glioblastoma stem cells. A) Kaplan‐Meier survival curve for all gliomas reported in the Cancer Genome Atlas (TCGA, N = 667). Green curve: HBEGF^low^, red curve HBEGF^high^ expression. B) Kaplan Meier survival curve for all gliomas reported in the Chinese Glioma Database (CGGA, N = 333). Green curve: HBEGF^low^, red curve HBEGF^high^ expression. C) Kaplan Meier survival curve for IDH wild type, mesenchymal‐like glioblastomas with HBEGF^low^ (green curve) and HBEGF^high^ (red curve) expression (CGGA, N = 53). D) HBEGF expression (log2 normalized counts) in TCGA and CCGA grade III astrocytomas and grade IV glioblastomas (N = 1000) including glioblastoma subtypes (boxed data points, N = 816). E) HBEGF expression (log2 normalized counts) in anatomically identified laser‐microdissected patient samples (Ivy‐GAP database, N = 122). F) UFK265 Human cortex spatial transcriptomics data of neoplastic cells (*VIM*, log2 gene expression) at the GB leading edge with the healthy brain cortex. Histological staining: hematoxylin/eosin (H&E, pink/purple). G) UFK265 Human cortex spatial transcriptomics data for *HBEGF* (log2 expression). White arrowheads highlight neoplastic cells as defined in panel F lining the tumor leading edge. Histological staining: hematoxylin/eosin (H&E, pink/purple). H) UFK248 Human GB spatial transcriptomics data of glioblastoma stem cells (log2 *CD44* gene expression). I) UFK248 Human GB spatial transcriptomics data of proliferating microvascular endothelium (log2 *PECAM1* expression). Histological staining: hematoxylin/eosin (H&E, pink/purple). J) UFK248 Human GB spatial transcriptomics data of HBEGF (log2 expression). Red frame highlights ho Histological staining: hematoxylin/eosin (H&E, pink/purple).

By altering HBEGF signaling in GB, HP‐NP induced the conversion of MES‐like GSCs into less plastic glial‐like cells, stopping resupply of new cancer stem cells and malignant propagation in the CNS. In brain tumor patients, HBEGF upregulation is consistently associated with shorter survival (TCGA and CGGA data, *N* = 1000, **Figure** **8**A,B). When filtering data to the most aggressive type of brain tumors, IDH^WT^ mesenchymal‐like glioblastomas, HBEGF upregulation is associated to halved survival time (*N* = 53, *p* = 0.0182, Figure 8C). HBEGF is upregulated in mesenchymal‐like glioblastoma, especially when compared to lower grade astrocytomas or glioblastomas from classical/astrocytic groups (Figure 8D). We explored patient databases compiling transcriptomic information from either microdissected histological sections (Ivy‐GAP, Figure 8E) and spatially resolved transcriptomics^[^ ^50^ ^]^ to verify HBEGF tissue distribution (Figure 8E–G). Consistent with our findings, HBEGF upregulation (approximately three‐fold) can be detected in stem‐like cell niches including microvascular and tumor leading edge, where plastic GSCs are typically found (Figure 8E). In spatial data, HBEGF hotspots are associated with tumor‐brain cortex interface (Figure 8F,G).

We further identified that MES‐like cells sheltered in the vicinity of PECAM1^+^ tumor vascular niches correlated with local HBEGF upregulation (Figure 8H–J; Figures S5 and S6, Supporting Information). This supported our ability to target HBEGF‐expressing glioma stem cell reservoirs using HP‐NP crossing the BBB. Moreover, aggressive cancers including leukemia and pancreatic adenocarcinomas exhibit significant upregulation of HBEGF (Figure S7, Supporting Information), warranting further investigations to assess HP‐NP clinical relevance beyond brain tumors.

## 3 Conclusion

GB remains a terminal disease with huge unmet medical need. Most success stories in oncology wards, including immunotherapies and precision medicine for peripheral cancers, usually fail to provide consistent therapeutic responses in GB. Our study identified HBEGF, a potent mitogen expressed on plasma membrane and key regulator of EGFR signaling, as an accessible target and an important player driving glioma phenotypic plasticity. Highly chemoresistant MES‐like cells, called *persister* GSCs sheltered in tumor vascular niche, are characterized by upregulation of four genes including HBEGF, target of HP‐NPs.^[^ ^51^ ^]^ We demonstrated that phenotypically stable GSCs expressing high levels of HBEGF^[^ ^52^ ^]^ sheltered in perivascular niches could be targeted by HP‐NP delivered systemically. HP‐NP selectively home to HBEGF‐expressing MES‐like GSCs, downregulate HBEGF expression and EGFR signaling, and directly interfere with GSC plasticity without need of cytotoxic drugs or chemotherapies. Our novel HP‐NP nanomaterials can be synthetized as sterile formulations, lyophilized, and stored stably at room temperature enabling clinical translatability. These HP‐NP can encapsulate different therapeutic payloads such as DOX and facilitate delivery across BBB. Toxicology studies in rats suggest HP‐NP are safe and well tolerated at therapeutic dose. In GB patient avatars, we demonstrate HP‐NP and HP‐DOX‐NP administered systemically can reach human GB cells progressing intracranially, however, therapeutic response was significantly higher for HP‐NP. Using comprehensive protein level and transcriptomics studies, we show HP‐NP not only reduced MES‐like GSC plastic abilities but also drove differentiation into less tumorigenic tumor cell states. We speculate HP‐NP could be used in clinical care as an adjuvant therapy to fragilize gliomas and prime tumor cells for classical chemotherapeutic regimen. Beyond brain tumors, we propose further investigations in other peripheral malignant cancers overexpressing HBEGF, which could exhibit sensitivity to HP‐NP.

## 4 Experimental Section

Unfractionated Heparin sodium salt (UFH) from porcine intestinal mucosa (cat # H3393‐50KU), Fluorescein‐5‐thiosemicarbazide (FTSC) (Prod # 46985‐100MG‐F), N‐hydroxybenzotriazole (HOBt) (Prod # 711489‐250G), 1‐ethyl‐3‐(3‐dimenthylaminopropyl) carbodiimide hydrochloride (EDC‐HCl) (Prod # E6383‐5G), and all solvents were purchased from Sigma‐Aldrich and used as received. Doxorubicin‐HCl (DOX) was purchased from Tocris Bioscience (Cat no. 2252). All solvents used were of analytical quality.

### HP‐NP and HP‐DOX‐NP Synthesis and Characterization

Fluorescein‐5‐Thiosemicarbazide (FTSC) conjugated HP‐NPs were synthesized using previously reported methods^[^ ^31^, ^53^ ^]^ by carbodiimide chemistry using HOBt and EDC. After synthesis, yellowish‐orange fluffy dry HP‐NPs were obtained following lyophilization and percentage of conjugation was estimated at 1.8% (with respect to disaccharide repeat units) using UV–vis spectroscopy on Shimadzu UV‐3600 Plus spectrophotometer with extinction coefficient of FTSC of 78 000 M^−1^cm^−1^ at 492 nm in phosphate buffer pH 7.4. Due to amphiphilicity of HP‐NPs that promotes nanoparticle self‐assembly in solution, H^1^‐NMR was not useful in determining conjugation %. Self‐assembly characteristics leads to disappearance or underestimation of hydrophobic molecules due to core‐shell assembly.^[^ ^31^ ^]^

DOX was loaded onto HP‐NPs via nanoprecipitation method as previously reported.^[^ ^34^ ^]^ In brief, 100 mg of HP‐NPs were dissolved in 25 mL of 1x PBS at pH 7.4 followed by dropwise addition of 5 mg of DOX‐HCl with constant stirring (1000 rpm). pH was readjusted back to 7.4, and mixture was stirred overnight. Reaction mixture was loaded into a dialysis bag (Spectra Por‐6, MWCO 3.5 kDa) and dialyzed against 2L of 100 mm NaCl with 2 water changes for 24 h followed by dialysis against deionized water for 24 h with 2x water changes. Dialysis under saline conditions was necessary to eliminate electrostatically bound DOX that were complexed with the carboxylates and sulfate groups of HP polymer, which cause burst release of the drug. Solution was lyophilized, and fluffy orange‐reddish HP‐DOX‐NP product was obtained with 96% yield.

### Quantification of DOX Loading

DOX loading was quantified by UV/vis spectroscopy at 485 nm. Briefly, 5 mg of HP‐DOX‐NPs was dispersed DMSO:PBS buffer (pH 7.4) in 1:1 ratio in a glass vial and was sonicated in water bath at 37 °C for 1 h to disrupt the self‐assembled particles. Subsequently, the solution was transferred to centrifugal filters (Amicon Ultra 4, regenerated cellulose membrane, 10 kDa MWCO, Sigma Aldrich Millipore, Cat #UFC801008D) and spun down at 4300 rpm for 10 min. The polymer in the cellulose membrane after first centrifugation was washed with DMSO:PBS mixture to release any residual DOX. The release solution was pooled and the absorbance of the release media (DMSO:PBS mixture) was measured by UV/vis spectroscopy at 485 nm (Lambda 35 UV/vis spectrometer, PerkinElmer) using the extinction coefficient of 11 500 M^−1^ cm^−1^. The % of DOX loading was estimated to be 4.6% w/w which corresponds to an encapsulation efficiency of 92% of drug loading.

The Drug Encapsulation Efficiency and Drug Loading were Determined by

(1) $$\begin{array}{cc} \mathrm{Encapsulation}\;\mathrm{efficiency}\;\left(\mathrm{EE}\right)\% & =\frac{\left(\mathrm{Weigh}t_{\mathrm{Total}\;\mathrm{DOX}}-\mathrm{Weigh}t_{\mathrm{Free}\;\mathrm{DOX}}\right)}{\mathrm{Weigh}t_{\mathrm{Total}\;\mathrm{DOX}}}\\ & \;\times 100 \end{array}$$

(2) $$\mathrm{Drug}\;\mathrm{loading}\;\left(\mathrm{DL}\right)\%=\frac{\left(\mathrm{Weigh}t_{\mathrm{Total}\;\mathrm{DOX}}-\mathrm{Weigh}t_{\mathrm{Free}\;\mathrm{DOX}}\right)}{\mathrm{Weigh}t_{\mathrm{HP}-\mathrm{NPs}}}\times 100$$

For the fluorescence measurements, the HP‐NPs and HP‐DOX‐NPs were resuspended in deionized water at 1 mg mL^−1^ and fluorometry emission spectra was evaluated from 515 to 600 nm using a QuantaMaster PTI Fluorometer.

### DOX Release Kinetics Experiment via Centrifugal Filtration

In vitro DOX release rate and stability analysis of HP‐DOX‐NP in 1xPBS pH 7.4 and in 25% fetal bovine serum (FBS) was performed by centrifugal filtration method. Briefly, DOX was dissolved at 0.046 mg mL^−1^ in 25% DMSO/PBS solution and 25% FBS/PBS solution (concentration matching 4.6 w/w% of DOX loaded onto HP‐DOX‐NP). 1 mg mL^−1^ HP‐DOX‐NP solutions were prepared in 1x PBS, 25% FBS in PBS, and in 25% DMSO in PBS as a positive control. Samples were incubated at 37 °C for the duration of the experiment. At each time point, 1 mL of each sample solution was pipetted into centrifugal filters (Amicon Ultra 4, regenerated cellulose membrane, 10 kDa MWCO, Sigma Aldrich Millipore, Cat #UFC801008D) and spun down at 4300 rpm for 10 min. The DOX concentration of the resulting fluid filtrate was analyzed using Tecan SPARK multimode plate reader measuring both UV–vis absorbance at 485 nm with an extinction coefficient of 11 500 M^−1^cm^−1^ followed by fluorescence spectroscopy with 470 nm excitation and 580 nm emission wavelengths. For max 100% DOX release baseline values, complete solutions without filtering were measured. All samples were covered with aluminum foil and protected from light to prevent DOX degradation throughout the experiment. Filtrates and leftover concentrates were remixed and placed back into sample tubes after each measurement time point without any loss. Background absorbance and fluorescence values were corrected with blank media wells (1x PBS, 25% FBS in PBS, 25% DMSO in PBS, and 25% FBS plus 25% DMSO in PBS). Experiment was repeated in triplicate with n = 3 replicates. % DOX release was calculated by the following equation:

(3) $$\%\mathrm{DOX}\;\mathrm{release}=\left(\frac{C_{t}}{C_{\mathrm{total}}}\right)*100\%$$

% *DOX* *release*  = (*C_t_* /*C_total_*)*100% where C_t_ is the concentration of DOX in the filtrate at time t and C_total_ is the total amount of DOX loaded in each sample formulation.

### Nanoparticle Size Analysis with Dynamic Light Scattering

Hydrodynamic particle size distribution and zeta potential of HP‐NPs and HP‐DOX‐NPs was determined with dynamic light scattering (DLS) using a Zetasizer Nano ZS (Malvern, UK) using 10 × 10 × 45 mm quartz cuvette. Lyophilized HP‐NPs and HP‐DOX‐NPs were resuspended in 1x PBS at 1 mg mL^−1^ and stirred for 15 min prior to running DLS analysis at 25 °C. Zeta potential analysis was conducted on HP‐NPs and HP‐DOX‐NPs resuspended in 1x PBS at 0.1 mg mL^−1^ in disposable 10 × 10 × 45 mm polystyrene cuvettes with Malvern Universal dip cell kit for zetapotential measurement.

### Scanning Electron Microscopy

Morphology of HP‐NP and HP‐DOX‐NP was characterized using scanning electron microscopy (SEM). Solutions of HP‐NP and HP‐NP‐DOX (1 mg mL^−1^ in PBS) were prepared and drop‐cast onto silicon wafers, followed by drying under an argon stream. Prior to imaging, a thin gold layer (≈2.5 nm) was sputter‐coated onto the sample surfaces. Scanning electron microscopy (SEM) was performed using a field emission gun SEM (FEG‐SEM, Zeiss Merlin) operated at an accelerating voltage of 3 kV. High‐resolution surface morphology was recorded using secondary electron (SE) detection with in‐lens annular detectors.

### Human Whole Blood Experiment

DOX, HP‐NPs, and HP‐DOX‐NPs were mixed into human whole blood using Chandler loop model to evaluate the hematological hemocompatibility. In brief, all the plastic materials including tubes, tips and containers were treated with heparin conjugates (Corline heparin, Corline Systems AB, Uppsala, Sweden) according to the company's protocol to avoid surface‐induced activation.

### In‐Vivo Safety Study

Two doses of HP‐NP were evaluated versus placebo in 24 RccHan:WIST rats (Envigo, Netherlands) of both sexes. Rats were eight weeks old, with a body weight of 197 ± 10 g (females) and 314 ± 25 g (males) at start of study (see Tables S1 and S2, Supporting Information for details on animals and housing). Rats were administered 2 mg kg^−1^ HP‐NP (1.2 mg mL^−1^), 20 mg kg^−1^ high dose HP‐NP (12 mg mL^−1^), or NaCl daily, via tail vein for 14 days. Concentration of high dose was decreased to 9 mg mL^−1^ on day 5 because rats had begun to struggle during injection. Dose volume was unchanged. Behavior was monitored daily, and body weight recorded every other day. On day 10–12, rats underwent ophthalmologic examination. During terminal anesthesia (day 15), blood was collected by cardiac puncture and tissue samples placed in formaldehyde. Plasma was extracted for analyses of hematology parameters (EDTA tubes), clinical chemistry (lithium heparin tubes), and coagulation parameters (citric acid tubes). Hematology and clinical chemistry parameters were analyzed at SLU (Clinical Chemistry Department). Prothrombin time (PT) and activated partial thromboplastin time (APTT) was analyzed by Adlego Biomedical, Uppsala, Diagnostica (Stago Start 4 Hemostasis Analyzer). Histology was performed in GLP compliant facility and to GLP standards by pathologist blinded to treatment (BioVet AB, Solna, Sweden). Brain, heart, kidney, lung, liver, and spleen from placebo (*n* = 4), 2 mg kg^−1^ (*n* = 4) and 10 mg kg^−1^ (*n* = 5) HP‐NP treated animal of both sexes were examined. Scoring of toxicity was performed according to Mann et al 2012 (see Supporting Information for details) on a scale from 0 (no changes) to 4 (severe changes).

### Antibodies and Reagents—Western Blotting

Monoclonal mouse anti‐EGFR (sc‐373746, Santa Cruz, 1:1000), monoclonal mouse anti‐HSC70 (sc‐7298, Santa Cruz, 1:1000), monoclonal mouse anti GFAP (G3893, Sigma‐Aldrich, 1:1000), polyclonal rabbit anti‐meteorin (600‐430, ThermoFisher, 1:500),  monoclonal mouse anti human vimentin (C9080, Sigma‐Aldrich, 1:1000), monoclonal mouse anti GFAP (G3893, Sigma‐Aldrich, 1:1000), and monoclonal mouse anti human HBEGF (sc‐74441, Santa Cruz, 1:1000).

### Antibodies and Reagents—Immunofluorescence

Monoclonal mouse anti‐human vimentin Cy3 conjugate (C9080, MilliporeSigma, 1:900); monoclonal rat anti‐mouse podocalyxin (MAB1556, R&D Systems, Bio‐Techne, 1:800) and mouse monoclonal anti human HBEGF (MAB2591, R&D Systems 1:200)

### Cell Culture

Isolation of patient‐derived GSCs (BT3, BT12, BT13, BT27, ZH305, and S24) was previously described.^[^ ^41^ ^]^ Patient‐derived glioma cell lines were maintained in serum‐free DMEM/F12 medium (BTs lines) or Neurobasal (ZH305, S24) supplemented with 1× B27 (both from Gibco, Thermo Fisher Scientific), 2 mm l‐glutamine, 100 U mL^−1^ penicillin, 100 µg mL^−1^ streptomycin, 15 mM HEPES (all from Lonza), 0.02 µg mL^−1^ human EGF, and 0.01 µg mL^−1^ human FGF‐basic (both from PeproTech).

### Western Blotting

Native patient avatar brain sections (8 to 12 per samples) on microscope slides were scraped and lysed in NP40 buffer containing protease and phosphatase inhibitor cocktails (Roche). Samples were lysed on ice for 30 min, sonicated, and microcentrifuged at 15000 RCF for 15 min at 4 °C. Protein concentrations were determined using the Lowry Protein Assay Kit (Bio‐Rad) according to manufacturer's instructions. Samples were boiled (95 °C, 5 min) in reducing Laemmli sample buffer, and 10 µg of protein per lane was separated on NuPAGE 4–12% precasted gels (NP0336BOX, Invitrogen). Proteins were transferred onto PVDF membranes using wet transfer sandwiches (Bio‐Rad). Membranes were blocked in 3% bovine serum albumin with 0.1% Tween‐20 (i.e., TBS‐T) for 1 h at room temperature and then incubated with primary antibodies overnight at 4 °C. After washes with 0.1% TBS‐T, membranes were incubated with appropriate HRP‐conjugated secondary antibodies (Bio‐Rad). Finally, signal was developed with either ECL or ECL+ substrates (ThermoFisher) and images acquired on a G:Box F3 system (Syngene).

### Hemocompatibility of Human Whole Blood Using ELISA Factor Xa Assay

Clotting activity of Heparin or HPNPs were evaluated by measuring factor Xa activity (BIOPHEN HEPARIN (AT+)) according to company's protocol. Samples and heparin were diluted with PBS to become 5 µg mL^−1^. Each well of 96 well plate was filled with each sample (15 µL). Next, antithrombin solution (from human, 15 µL) was added to each well and mixed, and substrate of FXa (Sxa‐11, 75 µL) was added into each well and incubated for 120 s. Then, solution of factor Xa (from bovine, 75 µL) was added into each well and incubated 90 s. Finally, 20 mg mL^−1^ citric acid solution (100 µL) was added to terminate the reaction. Absorbance at 405 nm of each well was detected with plate‐leader. All reactions were conducted at room temperature. Three different concentrations of UFH were used for the standard curve.

### Hemocompatibility Assay Using Blood Loop Model

Human whole blood was drawn into a collection container from a healthy donor who had received no medication at least 14 days before blood donation, and no anticoagulants were added. Immediately, 1 mL of whole blood was mixed with 60 µm Dox or Dox equivalent of HP‐Dox‐NPs (in PBS) and enclosed it in heparinized tubing coated with Corline heparin. Equal amounts of HP‐NPs (in PBS) and volumes of PBS were used as control. The tubing was closed with a Corline heparinized connector and rotated at 22 rpm for 1 h at 37 °C. The blood was subsequently taken out from the tubing and was carefully transferred to a tube containing EDTA solution (pH 7.4, final concentration: 10 mm). Platelet count was obtained for each sample using a Sysmex XP‐300 Automatic Hematology Analyzer (Sysmex Corporation, Kobe, Japan). Platelet number was expressed as remaining platelets in blood sample compared to initial value in blood prior to exposure to incubation vial for each of blood models (set to 100%) and are given as mean percent ± SD. After blood was centrifuged at 2500 x g for 15 min at 4 °C, plasma sample was collected and stored at −80 °C before analyzing concentrations of complement markers (C3a and soluble C5b‐9 complex (sC5b‐9)) and thrombin‐antithrombin complexes (TAT) (https://doi.org/10.1039/c5cc09215a). Factor XII (FXII) and Factor XI (FXI) were measured, Antithrombin (AT) was the most important inhibitors that form complexes with FXII and FXI. The formation of FXIIa–AT complex and FXIa–AT complex was believed to be specific for clotting and fibrin generation. Here FXIIa–AT, FXIa–AT complex levels wERE measured when these samples were added into blood.

### Hemolysis Assay in Human Donor Blood

Nine milliliters of human whole blood was drawn into sodium citrate stabilized tubes from 3 different healthy donors procured from the Finnish Red Cross Blood Service at room temperature. Hemolysis assay was conducted on the same day as blood collection within 3 h after blood draw. Briefly, 3 mL of blood from each donor was centrifuged at 1600 rpm for 5 min and blood plasma and surface layers were gently removed. The remaining RBC pellets were washed and spun down 5 times with at least 6 mL of PBS each time. The remaining washed RBC pellets were resuspended in 7 mL of PBS vehicle. 0.8 mL of samples were prepared in PBS vehicle in triplicate and 0.2 mL of RBC solution was added for a final volume of 1 mL per sample. Sample groups included 0.1 mg mL^−1^ HP‐NP, 0.1 mg mL^−1^ HP‐DOX‐NP, 5 µg mL^−1^ DOX, and 20 µg mL^−1^ DOX. Positive control was prepared with 0.8 mL deionized H_2_O and 0.8 mL PBS vehicle was used as negative control. The samples were incubated at room temperature for 2 h on a gentle orbital rocker set to 40 rpm. Samples were additionally gently shaken every 30 min to resuspend any settled RBCs and NPs. After 2 h, samples were centrifuged at 1600 rpm for 5 min and 100 µL of supernatant from each sample were transferred to 96 well plates. Absorbance of hemoglobin in supernatants was measured with TECAN Spark microplate reader at 541 nm. Hemolysis percentage of RBCs was calculated with the following formula:

(4) $$\%\;\mathrm{Hemolysis}=\frac{\left(\mathrm{Abs}.\;\mathrm{Sample}\;-\mathrm{Abs}.\mathrm{Neg}.\mathrm{Control}\right)}{\mathrm{Abs}.\;\mathrm{Positive}\;\mathrm{Control}-\mathrm{Abs}.\mathrm{Neg}.\mathrm{Control}}\cdot 100\%$$

### HP‐NP Uptake Study and Flow Cytometry

For flow cytometry HP‐NP, HP‐DOX‐NP, and DOX uptake assay, 100 × 10^3^ cells were plated in 5 mL flasks with each respective condition and for each patient derived GSCs (BT3, BT12, and BT27). Groups were untreated control cells in GSC complete cell media incubated at 37 °C, 0.1 mg mL^−1^ HP‐NP resuspended in GSC media incubated at 37 °C for 2 h, and 0.1 mg mL^−1^ HP‐NP incubated at 4 °C for 2 h. Identical groups were tested with HP‐DOX‐NPs in parallel. For DOX uptake groups, DOX was initially dissolved into deionized H_2_O at 5 mg mL^−1^ and diluted into complete GSC media to a final concentration of 4.6 µg mL^−1^. DOX groups included all BT lines exposed to 5 µg mL^−1^ DOX in GSC media incubated at 37 °C for 2 h and 4.6 µg mL^−1^ DOX in GSC media incubated at 4 °C for 2 h in addition to untreated controls. Following respective condition exposure, GSC cells were removed from flasks, pelleted by spin down at 1200 rpm for 5 min, washed with cold 1xPBS, pelleted again by spin down, and resuspended in 200 µL accutase (Gibco StemPro Accutase Cell Dissociation Reagent, Thermofisher Catalogue Number A111050) and incubated for 5 min at 37 °C. Following accutase incubation, cells were washed with cold 1xPBS, resuspended in 1xPBS with 1 µg mL^−1^ 4′,6‐diamidino‐2‐phenylindole (DAPI) (Thermofisher Scientific, Cat # D1306) and incubated for 5 min on ice. The cells were spun down and washed again with cold 1x PBS and subsequently resuspended in 200 µL cold 1xPBS and placed on ice prior to FACS. Cell suspensions were analyzed using CytoFlex S Flow Cytometer (Beckman Coulter) followed by gating and analysis in FlowJo software package (FlowJo LLC). 10000 events were captured from each sample and experiments were repeated in triplicate. Single cell populations were isolated using gating on forward and side scatter density plots, first with side scatter area (SSC‐A) over forward scatter area (FSC‐A) followed by forward scatter height (FSC‐H) by FSC‐A. Cell viability was established from single cell populations using DAPI staining gates in PB450 channel against SSC‐A. Cells staining positive for DAPI compared to control cell grouping were excluded as dead cells with damaged cell membranes. Control cells were used to create gates and establish live/dead viability across all conditions. FITC positivity was further analyzed from the live cell population (negative PB450) gates, and untreated GSC control samples was used to establish FITC positivity in SSC‐A versus FITC‐A and FITC‐A histogram plots. See Figure S3 (Supporting Information) for example gating strategy data on all cell lines used in Control and HP‐NP treated groups.

### Proliferation and Cell Viability Assay

For cell proliferation and viability assays, 10 × 10^3^ cells were plated in triplicates in 96‐well plates in complete culture media with growth factors. Cells in test groups were exposed to HP‐NP, HP‐DOX‐NP, UFH, and DOX suspended in complete culture media. After 48 h of incubation, cells were treated with 10 µL of 3‐(4,5‐dimethylthiazol‐2‐yl)‐2,5‐diphenyltetrazolium bromide (MTT; 5 mg mL^−1^ in PBS) and incubated for 4 h at 37 °C in 5% CO_2_. Cells were lysed in 10% SDS‐10 mm HCl and incubated further overnight at 37 °C, and absorbance was measured at 540 nm using a Viktor Nivo multimode microplate reader (Perkin Elmer). IC50 values were estimated from cytotoxicity data using absolute IC50 non‐linear regression fitting in GraphPad Prism software. Experiments were repeated at least 3 times.

### RT‐qPCR and mRNA Sequencing Following HP‐NP Exposure In Vitro

For RT‐qPCR and mRNA sequencing of in vitro BT12 GSCs following HP‐NP exposure, 20 × 10^4^ BT12 GSC cells were plated in T25 flasks in each exposure condition using complete GSC media with growth factors and incubated at 37 °C for 24 h prior to cell lysis and RNA extraction. The treatment conditions were as follows: untreated BT12 cells in complete GSC media were used for controls, 0.5 mg mL^−1^ UFH in complete GSC media, 0.5 mg mL^−1^ HP‐NP in complete GSC media, 0.5 mg mL^−1^, and 0.25 mg mL^−1^ HP‐DOX‐NP in complete GSC media, and 25 µg mL^−1^ free DOX in complete GSC media. Treatment condition concentrations were chosen based on the estimated IC50 values to ensure cell survivability over 24 h incubation, especially in DOX containing groups. Following 24 h incubation, cell media was removed and Qiagen RNA cell protect reagent was added followed by RNA extraction using Qiagen's RNAEasy Mini Plus kit. RNA concentration and purity was estimated using Thermo Scientific Nanodrop One Spectrophotometer (Thermofisher scientific).

For RT‐qPCR, RNA samples were converted to cDNA using Applied Biosystems’ High capacity cDNA reverse transcription kit (Thermofisher Scientific, catalogue number 4 368 814). Concentration of RNA was set equal across all treatment conditions to 10 ng µL^−1^ in cDNA conversion step. qPCR was conducted with TaqMan Probes (Thermofisher Scientific) and Taqman Fast Advanced Master Mix (Applied Biosystems, catalogue #4 444 557). Taqman Probes used included GAPDH human (Assay ID Hs02786624_g1), ACTB human (Assay ID Hs01060665_g1), HBEGF Human (Assay ID Hs00181813_m1), and VEGFa Human (Assay ID Hs00900055_m1). Fold change was quantified using log 2 ΔΔ Ct method normalized to GAPDH expression and compared to untreated BT12 control group. ACTB was used as a secondary housekeeping gene, and results remain unchanged when using ACTB as housekeeping gene.

RNA samples were shipped on dry ice to Novogene for mRNA Sequencing. mRNA sequencing was performed by Novogene using Illumina NovaSeq platform. Raw mRNA read data was quality checked by Novogene and as a secondary with FastQC.^[^ ^54^ ^]^ Raw reads were aligned to reference human genome (Homo Sapiens GRCh38.110) provided by NCBI database^[^ ^55^ ^]^ using STAR alignment.^[^ ^56^ ^]^ Read counts matrices were produced from aligned datasets via featureCounts.^[^ ^57^ ^]^ Further downstream analysis, data processing, and TPM normalization steps was conducted in R Studio using R and Bioconductor packages.^[^ ^58^ ^]^ Packages used in data processing steps included edgeR,^[^ ^59^, ^60^, ^61^ ^]^ limma,^[^ ^62^ ^]^ Glimma,^[^ ^63^ ^]^ and GEOquery^[^ ^64^ ^]^ for converting gene labels.

### Preclinical Studies—Patient Avatars: Intracranial Orthotopic Tumor Xenografts

Intracranial implantation of patient‐derived gliospheres was performed as described.^[^ ^41^ ^]^ Briefly, 6‐week‐old Rj:NMRI‐Foxn1nu/Foxn1nu (Janvier Labs) female mice housed in cages of 4 and fed ad libitum were placed under 2.5% isoflurane anesthesia on a stereotaxic injector and intracranially engrafted with 1 × 10^5^ cells (in 5 µL PBS) obtained from dissociated gliospheres at the following distance from the bregma: +1 mm anteroposterior, +2 mm right, +2.5 mm depth. Postoperative analgesia (temgesic) was locally administered for 2 days.

### Preclinical Studies—Therapeutic Regimens

Following twelve days of recovery and tumor initiation, patient avatars were treated thrice a week for two weeks through left caudal vein infusion (6 µL s^−1^, 200 µL total) using sterile 27‐G Myjector insulin needle. Treatment groups included vehicle saline (*n* = 3), doxorubicin (n = 3, 1.5 mg kg^−1^), heparin nanoparticles (HP‐NP, n = 6, 5 mg kg^−1^) or doxorubicin‐loaded heparin nanoparticles (HP‐DOX‐NP, n = 6, 5 mg kg^−1^).

### Preclinical Studies—In Vivo Bioluminescence Imaging

Twice a week, patient avatars were briefly anesthetized (3% isoflurane), injected intraperitoneally with d‐luciferin (Sigma‐Aldrich, 15 mg mL^−1^ in PBS) at a dose of 10 µL per gram of body weight, and allow for systemic diffusion for 5 min before imaging. Up to 5 avatars were simultaneously placed under the gas mask anesthesia (1.8–2% isoflurane) of the bioluminescence imager (Lago‐Spectral Instruments Imaging, Bruker). For weekly image acquisitions, field of view was set from 10 to 15 (depending on the number of imaged avatars), Binning and FStop to 4 (early tumor growth timepoint) to 16 (later timepoints where tumors generated higher bioluminescence). Raw photon counts were determined using the ROI tool in Aura in vivo Imaging Software (Spectral Instruments Imaging, Bruker).

### Preclinical Studies—Ethical Endpoint and Tissue Collection

Avatars’ weight was measured every day and signs of tumor progression (e.g., >10% weight loss, hemiplegia) were carefully monitored. At the end of the experiment, animals were briefly anesthetized (3% isoflurane) and euthanized by cervical dislocation. Brains were snap‐frozen in −50 °C isopentane (Honeywell) until tissue processing.

### Immunofluorescence Staining of Tumor Tissue Xenografts

Snap‐frozen xenografted brains were cut, from frontal to the posterior part of diencephalon, using a cryotome (Leica CM3050) into 9 µm thick coronal sections serried on microscope slides. Before immunofluorescence staining, brain sections were fixed in 4% PFA and blocked with 5% FBS and 0.03% Triton X‐100 (MilliporeSigma). Whole slides were scanned using slide scanner (3DHistech). Tumor volumes were calculated using the histological coordinates defined by the brain section series. Histology quantification (e.g., tumor cell invasion, blood vessel density) was performed on 10 sections equally distributed along the entire tumor, for every patient avatar.

### Tumor Necrosis Quantification

Necrotic area annotation and quantification was performed on two digitalized histological sections per animal in all cohorts. Briefly, the *closed polygon* annotation tool in Slideviewer 2.7 (3DHistech) was used to manually delineate tumor necrotic areas, based on DAPI nuclear counterstain features. Values were exported using the annotation export feature and analyzed in Graphpad Prism.

### Tumor Invasion Quantification

Cell annotation and distance measurement was performed on one histological section of three animals per cohort. The *draw linear measurement annotation* tool in Slideviewer 2.7 (3DHistech) was used to manually measure individual distances between the border of the main tumor mass and cell satellites, identified by the anti‐human vimentin cell immunofluorescence. Raw data were exported using the annotation export feature and analyzed in Graphpad Prism.

### Microvascular Endothelium Area Quantification

Intratumoral microvascular endothelium number and area quantification was performed on three animals per group. Briefly, 2.5x magnification captures including PODXL immunofluorescence labeling of the tumor microvascular endothelium was exported from Slideviewer 2.7 (3DHistech) to Fiji 1.54p (NIH). Images were first processed into binary files using the *make binary* function, and blood vessel size and number was determined using the *analyse particle* function set to detect elements included within 0‐infinity µm^2^. Raw data were exported as .csv for data curation and analyses, with element <180 µm^2^ excluded from the analyses as deemed unspecific/autofluorescent counts. Data was then analyzed for statistical significance in Graphpad Prism 10.

### Microscope Imaging

Fluorescence micrographs were acquired on EVOS microscopes (Invitrogen) with appropriate light sources. Whole slides were digitalized at the Genome Biology Unit (University of Helsinki, Finland) using a Pannoramic SCAN II Digital Scanner (3DHistech).

### Transcriptomics—Total RNA Re‐Analyses

Some data discussed in this publication (Figure S1, Supporting Information) were deposited in NCBI's Gene Expression Omnibus (GEO)^[^ ^65^ ^]^ and were accessible through GEO Series accession number GSE169418 (https://www.ncbi.nlm.nih.gov/geo/query/acc.cgi?acc = GSE169418).

The *z* scores were calculated as: reads of each sample – mean of all samples/SD. For phenotypic state profiling,^[^ ^1^ ^]^ list of genes for given signature was retrieved from original publication, and individual *z* scores were calculated among all samples.

### Transcriptomics—Publicly Available Databases

Normalized raw counts of patient glioma RNA sequencing data generated by TCGA Research Network (https://www.cancer.gov/tcga), CGGA^[^ ^66^ ^]^ (http://www.cgga.org.cn/) and IVY Glioblastoma Atlas Project (https://glioblastoma.alleninstitute.org/) was retrieved from GlioVis^[^ ^67^ ^]^ Human pan‐normal, and cancer data from TCGA Research Network and Genotype Tissue Expression (https://gtexportal.org/) was retrieved from Gene Expression Profiling Interactive Analysis portal (http://gepia2.cancer‐pku.cn).

### Transcriptomics—Spatial Transcriptomics Re‐Analyses of Human Glioblastoma Samples

Raw count matrices with corresponding histological section images and spatial coordinates were retrieved from dataset included in Ravi et al.’s 2022 publication.^[^ ^50^, ^68^ ^]^ 25 datasets were imported to Chipster 2,^[^ ^69^ ^]^ running *Seurat* v5 for quality control, preprocessing, spots clustering and gene expression visualization (Panel A). Low quality spots were filtered out based on high mitochondrial (>22%), low ribosomal (<2%), and high hemoglobin (>20%) transcript percentage. Twenty‐two samples passed quality control, leaving out UFK256 (tumor core) UFK262 (tumor), and UFK265 (tumor) from downstream analyses due to high mtRNA (>70%) and low nCount and nFeature per spot (<50). Data integration anchors were calculated using 50 dimensions and 22 datasets were then integrated into single normalized object using IntegrateData()‐function. Next, principal component analysis was run, and *Seurat* clusters were calculated using 30 dimensions and resolution of 0.8 using the FindClusters()‐function. Calculated clusters were visualized using Uniform Manifold Approximation and Projection (UMAP) and labelled based on differentially expressed genes (DEGs) retrieved using the FindMarkers()‐function. VisualizeGeneExpression()‐function was then used to determine spatial coordinates and gene expression levels for genes of interest (HBEGF), neoplastic cells markers (vimentin^high^), microvascular cerebral endothelium (PECAM1) and mesenchymal‐like glioblastoma cells (CD44 + vimentin^high^).

### Preclinical Safety Assessment in Rats

Eight‐week‐old RccHan: WIST rats (Envigo, the Netherlands), females *n* = 12, males *n* = 12, free of pathogens according to FELASA recommendations for health monitoring^[^ ^70^ ^]^ were brought to laboratory animal facility at SLU, Uppsala, Sweden. Animals were housed in groups of six in open cages (Rabbit Cage EC3, Scanbur, Karlslunde, Denmark). Animals had free access to feed (R36, Lantmännen, Stockholm, Sweden) and water. Temperature was kept at 20–24 °C and a 12:12: h light:dark schedule was applied, lights on at 6AM. After five days of habituation, rats were trained to get accustomed to handling and restraint for intravenous injection. Each rat was handled 2 min per day for 14 days. During training, rats were placed on a piece of fabric on a table. Trainer gently held and handled each rat and fixated the tail with one hand. After training or blood sampling, each rat was rewarded with edible treats and by being placed in a large enclosure with structural enrichment.

Daily intravenous experimental injections were given in either tail veins through a peripheral venous catheter (BD Neoflon 26G, BD, Franklin Lakes, NJ, USA) to ensure venous access before injection. On treatment day 8–10, two mL of blood was sampled from 6 rats on treatment day 8–10 for diagnostic purpose. Unfortunately, these samples were lost due to technical error. During terminal anesthesia (day 15), blood was collected by cardiac puncture under isoflurane anesthesia and tissue samples collected and placed in formaldehyde. Plasma was extracted for analyses of hematology (EDTA tubes), clinical chemistry (lithium heparin tubes), and coagulation parameters (citric acid tubes). Hematology and clinical chemistry parameters were analyzed at SLU (Clinical Chemistry Department). Prothrombin time (PT) and activated partial thromboplastin time (APTT) was analyzed by Adlego Biomedical, Uppsala, Diagnostica (Stago Start 4 Hemostasis Analyzer, analysis validated for rodents).

Histology was performed in a GLP compliant facility and to GLP standards by pathologist blinded to treatment (BioVet AB, Solna, Sweden). The brain, heart, kidney, lung, liver, and spleen from placebo (*n* = 4), 2 mg kg^−1^ (*n* = 4), and 20 mg kg^−1^ (*n* = 5) HP‐NP treated animal of both sexes were examined. Scoring of toxicity was performed according to Mann et al 2012 on a scale from 0 (no changes) to 4 (severe changes).^[^ ^71^ ^]^

### Statistics

All statistics were computed using GraphPad Prism versions 8 and 10 or InVivoStat v4.02 (Bate, S.T. and Clark, R.A. 2014). Statistical significance between sample groups was determined using unpaired 2‐tailed *t* test or nonparametric Mann‐Whitney *U* test. For multiple comparisons, 1‐way ANOVA with Kruskal‐Wallis test, or 2‐way ANOVA with Sidak's or Tukey's multiple comparisons tests, were used. Data were presented as mean ± SD or ± SEM, and *P* < 0.05 was considered statistically significant. Statistical tests were specified on Figure panels and/or in Figure legends. All images and graphs shown were repeated iterations or representative of several experiments as indicated in Figure legends.

### Ethical Statements

The use of human glioma tissue biopsies was approved by the Ethics Committee of the Northern Savo Hospital District (53/2009; 1.8.2009‐31.7.2019, Kuopio, Finland). All patients gave their written informed consent. Ethical approval for human whole blood experiments were obtained from the regional ethics board (Uppsala diary number 2008/264). Human donor blood for non‐clinical use was obtained from the Finnish Red Cross Blood Service under permit # 52/2024, Tampere University. Animal experiments were approved by either the Committee for Animal Experiments of the District of Southern Finland (ESAVI/6285/04.10.07/2014 and ESAVI/403/2019) or the Uppsala Committee of Animal Research Ethics (5.8.18‐00055/2021).

## Conflict of Interest

The authors declare no conflict of interest.

## Supporting information



Supporting Information

Supporting Information

## Data Availability

The data that support the findings of this study are available from the corresponding author upon reasonable request.
