# Supplementary material for: Therapeutic Reprogramming of Glioblastoma Phenotypic States Using Multifunctional Heparin Nanoparticles
Source: Adv Sci (Weinh). 2025 Nov 3;13(3):e09590. doi: 10.1002/advs.202509590 (PMC12806396; doi:10.1002/advs.202509590)
Supplement: Supplementary file 1 — Supporting Information [file ADVS-13-e09590-s002.docx]

**Supplementary Materials**

**
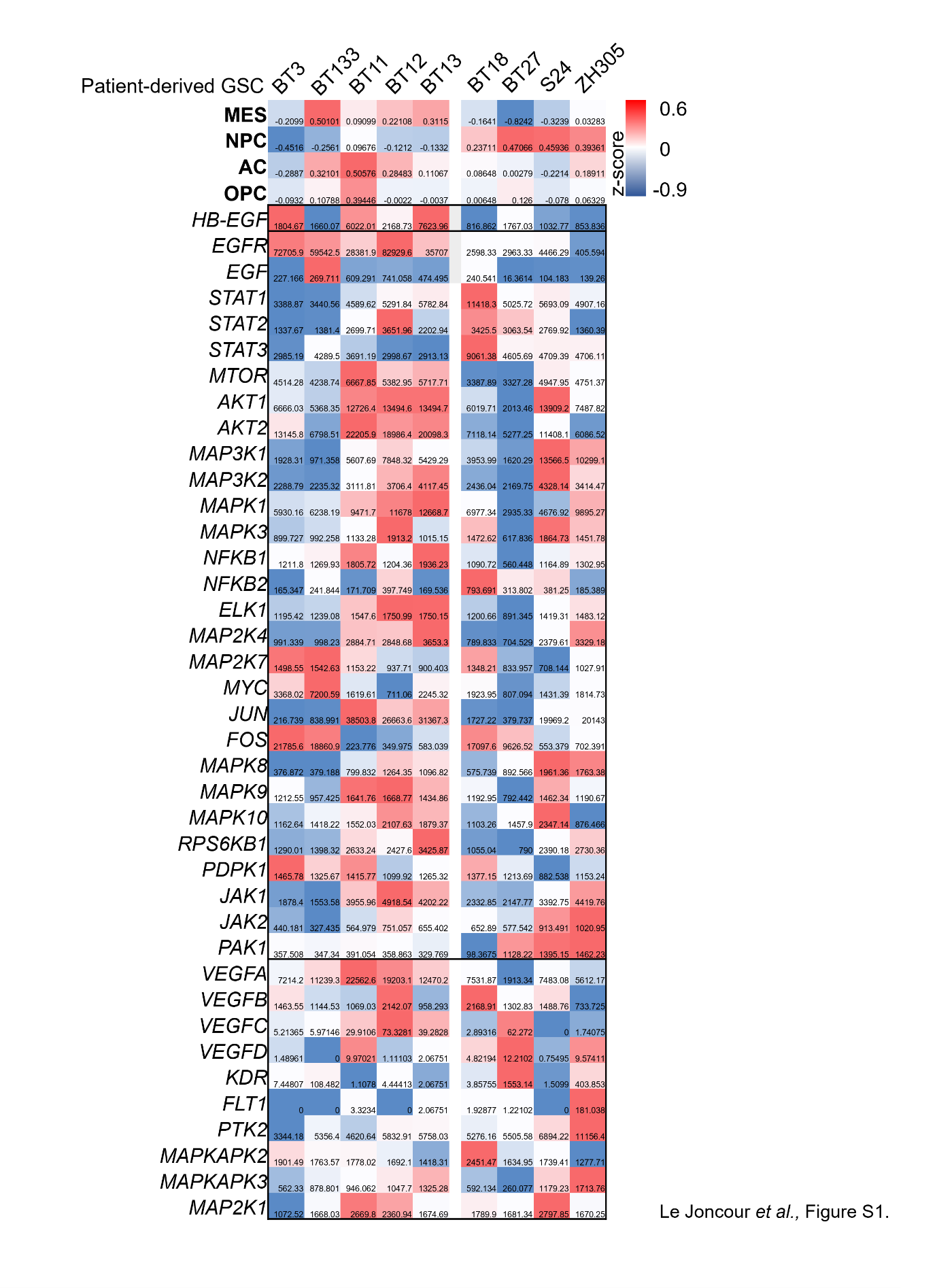
**

**Supplementary Figure S1: Patient-derived phenotypic cell states and resting state gene expression profiles for the HBEGF/EGFR and VEGFRs signaling pathway markers.** Top, Z-scores for the mesenchymal (MES), neuron precursor cell-like (NPC), astrocyte-like (AC) and oligodendrocyte precursor-like (OPC) phenotypic cell states calculated from aggregated normalized raw count values for the gene expression signatures established by Neftel et al., 2019. Bottom, normalized transcript per million values for the annotated genes. Re-analyzed total RNA sequencing data from GSE169418 (Filppu et al., 2021).

**Supplementary Table S1: FACS uptake experimental values** of BT3, BT12, and BT27 GSC cells in all conditions after gating for single live cells. 10,000 events captured for all groups.

|  | **BT3** | | | | **BT12** | | | | **BT27** | | | |
| --- | --- | --- | --- | --- | --- | --- | --- | --- | --- | --- | --- | --- |
|  | **Live cell Count** | **% Uptake** | **Mean FITC MFI** | **FITC MFI SD** | **Live Cell Count** | **% Uptake** | **Mean FITC MFI** | **FITC MFI SD** | **Live Cell Count** | **% Uptake** | **Mean FITC MFI** | **FITC MFI SD** |
| **HP-NP** | 6631 | 88.9 | 56209 | 345174 | 7523 | 97.3 | 74862 | 39841 | 3970 | 96.8 | 212185 | 364419 |
| **HP-NP 4°C** | 7409 | 13.4 | 18860 | 43787 | 7605 | 9.91 | 18682 | 27395 | 6391 | 34.8 | 53933 | 156131 |
| **HP-DOX-NP** | 6868 | 91.3 | 48484 | 159502 | 7484 | 98.6 | 67966 | 31190 | 4338 | 99.2 | 183092 | 4.56E5 |
| **HP-DOX-NP 4°C** | 7098 | 25.2 | 26105 | 139309 | 7780 | 22.5 | 24128 | 58293 | 6804 | 36.8 | 64208 | 356930 |
| **DOX** | 3144 | 73.4 | 36854 | 18068 | 3685 | 99.4 | 80317 | 31935 | 3733 | 97.5 | 100936 | 50343 |
| **DOX 4°C** | 4359 | 26.6 | 20239 | 10417 | 3662 | 40.5 | 27555 | 12703 | 6362 | 43.3 | 37015 | 19201 |
| **Control** | 6421 | 0.031 | 4401 | 2154 | 6756 | 0.030 | 8197 | 3273 | 5387 | 0.062 | 7868 | 12700 |


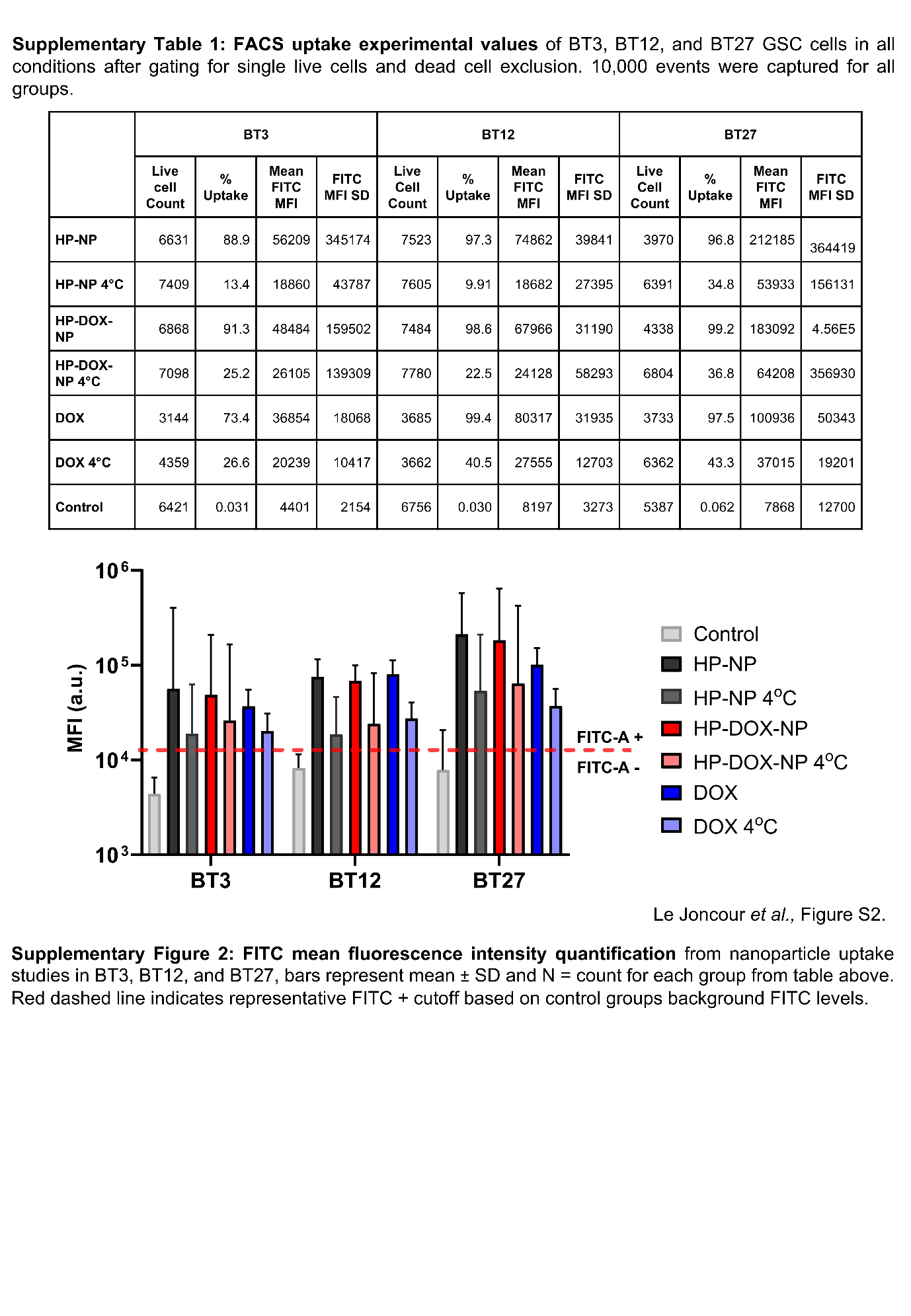


**Supplementary Figure S2: FITC mean fluorescence intensity quantification** from nanoparticle uptake studies in BT3, BT12, and BT27, bars represent mean ± SD and N = count for each group from table S1 above. Red dashed line indicates representative FITC + cutoff based on control groups background FITC levels.


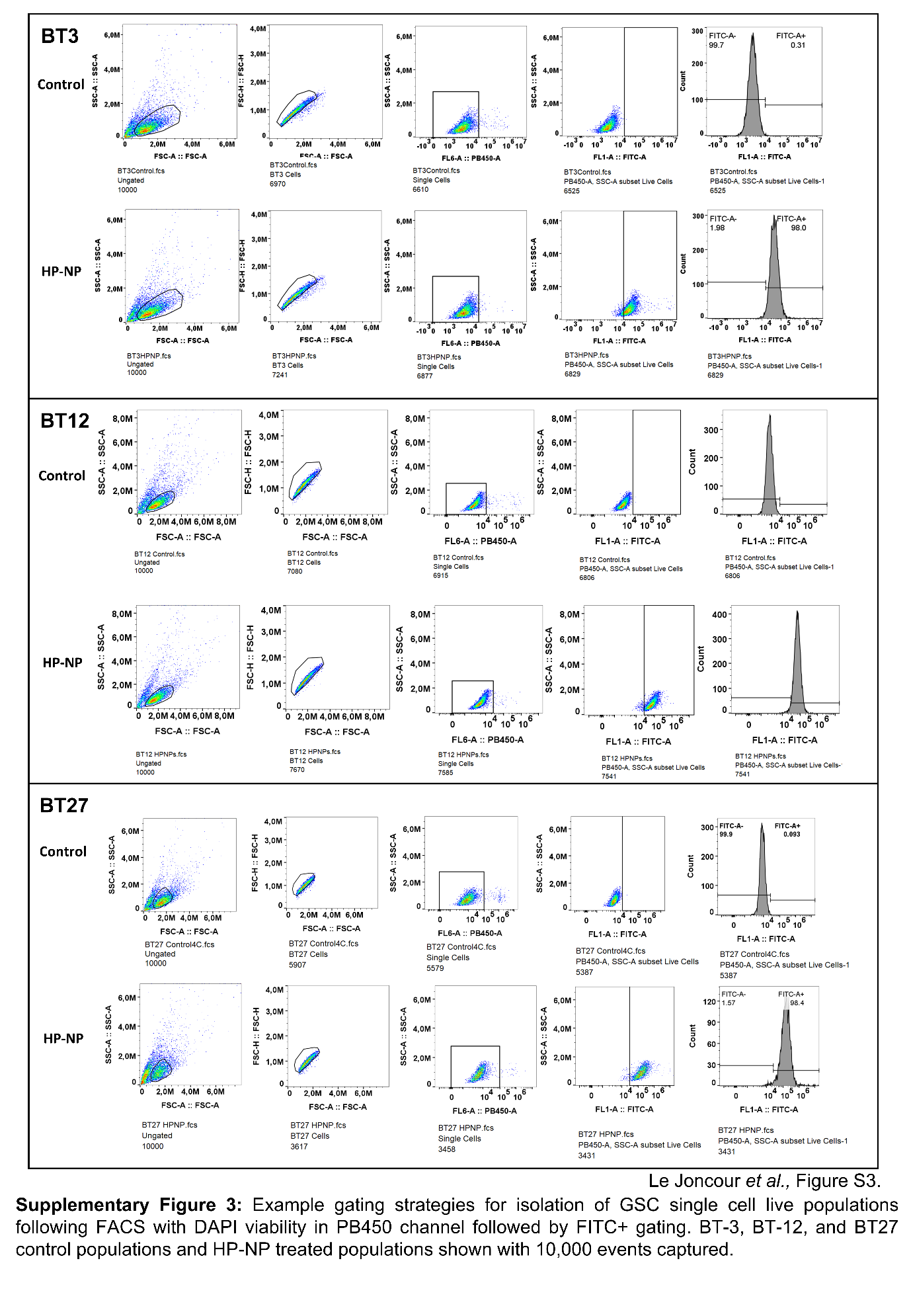


**Supplementary Figure S3:** Example gating strategies for isolation of GSC single cell live populations following FACS with DAPI viability in PB450 channel followed by FITC+ gating. BT-3, BT-12, and BT27 control populations and HP-NP treated populations shown with 10,000 events captured.


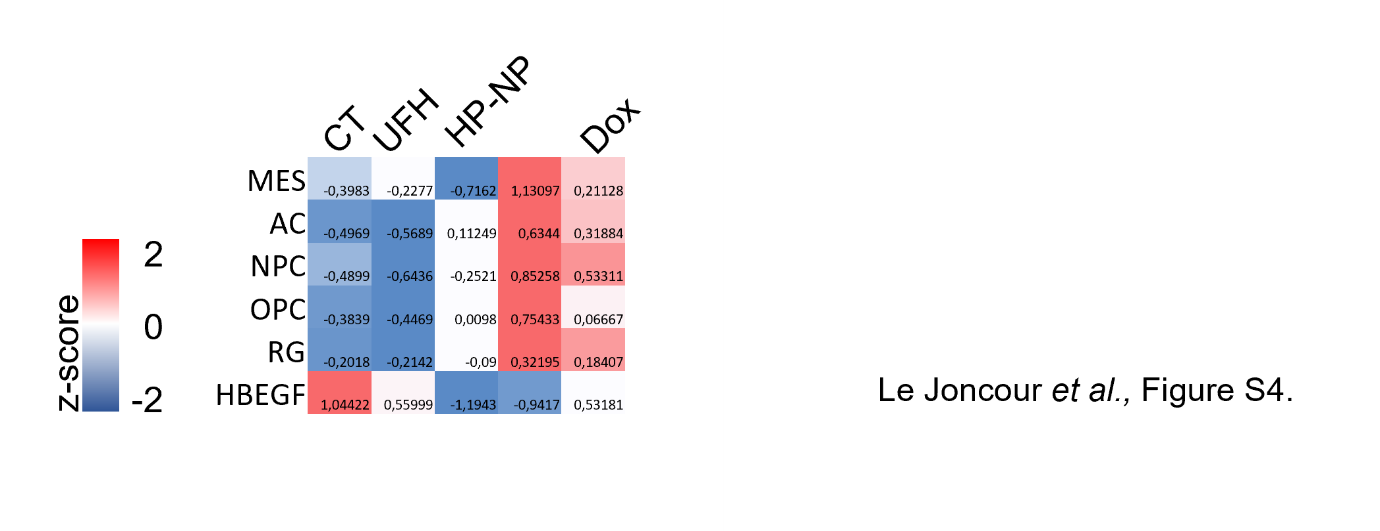


**Supplementary Figure S4: HP-NPs induces glioma stem cell differentiation and phenotypic cell state shift from mesenchymal-like to astrocyte-like glioblastoma. A**, Gene expression profiling of phenotypic cell states dynamics in control (CT), UFH, HP-NP, HP-DOX-NP and DOX treated BT12 GSCs (24h). Gene expression signature according to Neftel et al., 2019. Z-scores calculated with normalized raw count values from bulk RNA sequencing. MES, mesenchymal-like, AC, astrocyte-like, NPC, neuron precursor-like, OPC, oligodendroglial-like, RG, radial glia-like.


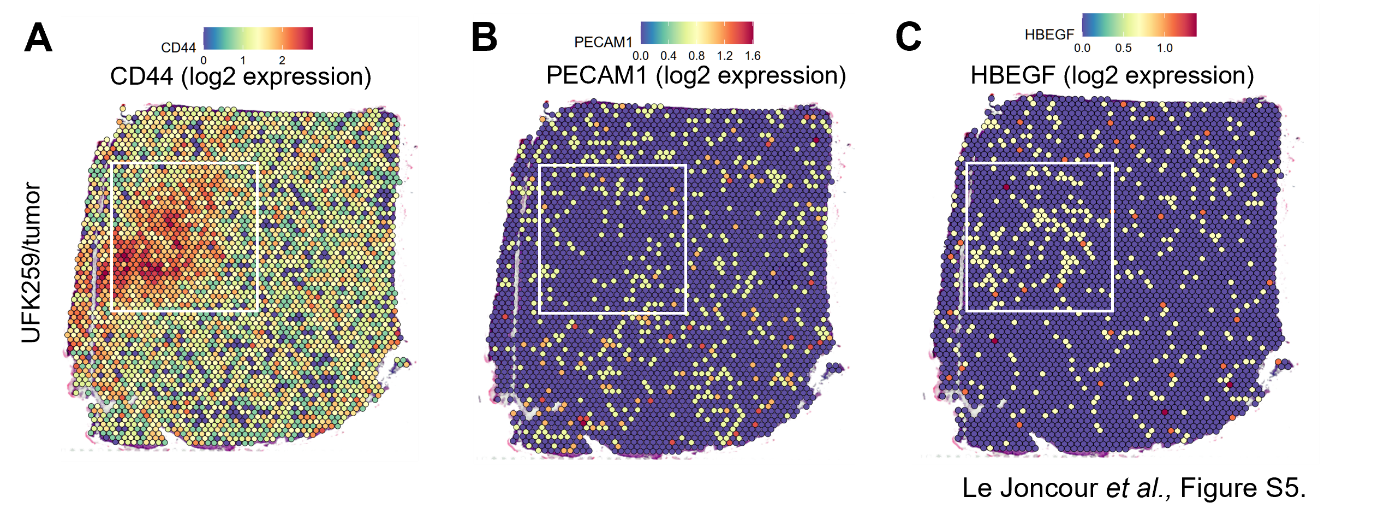


**Supplementary Figure S5: *Addendum* on HBEGF is a patient biomarker of fast progressing mesenchymal-like glioblastoma stem cells. A**, Human GB spatial transcriptomics data of glioblastoma stem cells (CD44, left panel), proliferating microvascular endothelium (PECAM1, middle panel) and hotspots of HBEGF expressing MES-like GB cells in vascular niches (right panel, red insert). Histological staining: hematoxylin/eosin (H&E, pink/purple).


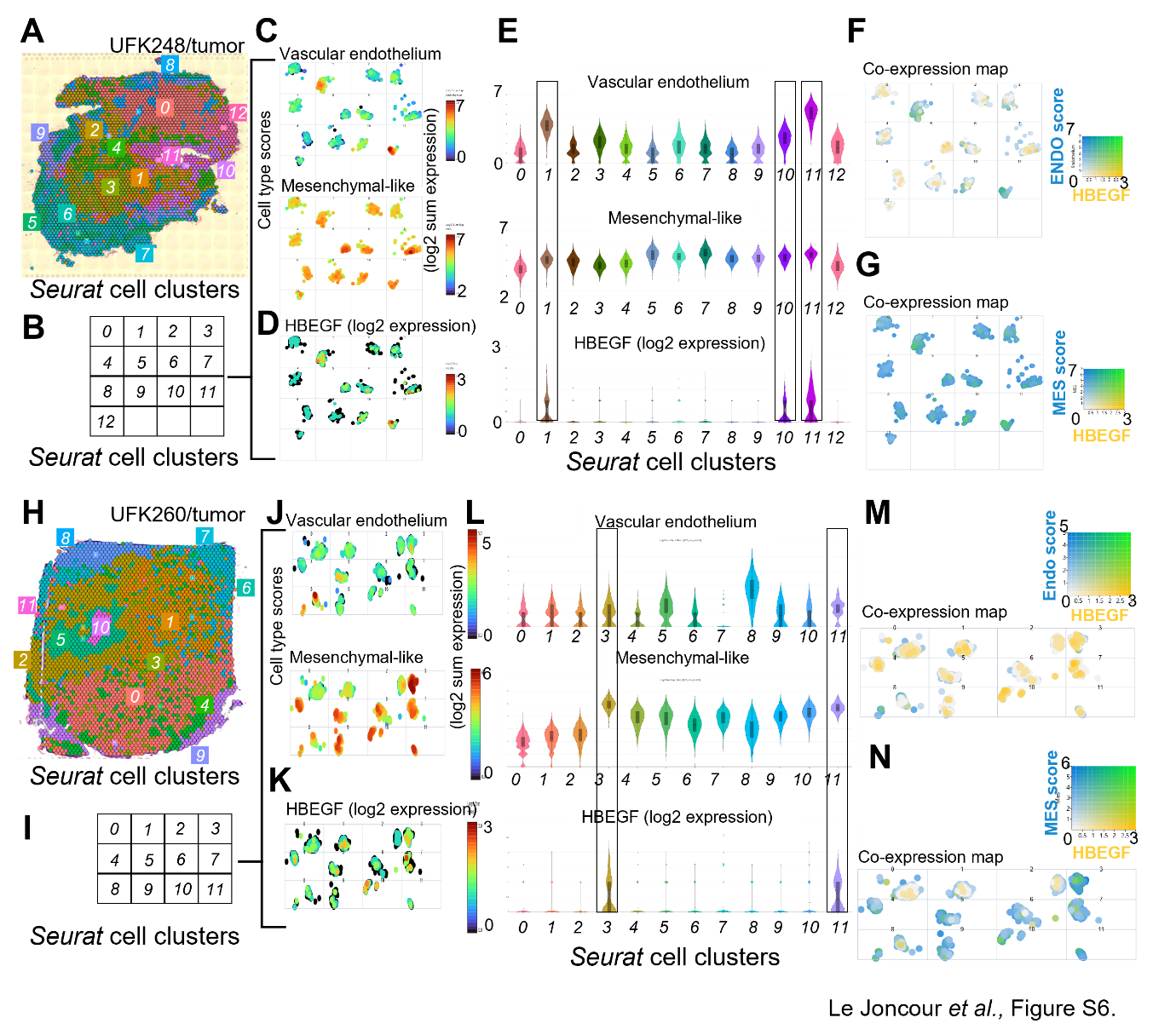


**Supplementary Figure S6: Extended data on the spatial distribution of HBEGF in tumour territories enriched with microvascular niches and mesenchymal-like glioblastoma stem cells. A**, *Seurat* Human GB cell clustering of UFK248 core GBM biopsy. Histological section stained with hematoxylin/eosin (H&E, pink/purple). **B**, *Seurat* cell labels from panel A as presented in panels C-D. **C**, vascular endothelium (top, includes *PECAM1*, *PODXL* and *COL4A1*) and mesenchymal-like (bottom, includes *CD44*, *VIM*, *P4HA1* and *HIF1A*) gene expression scores superimposed on the Universal Manifold Approximation Projection 2 (UMAP2) plots of the *Seurat* clusters as represented in A-B. Score expressed as sum of log2 gene expression. **D**, HBEGF gene expression (log2 expression) superimposed on the Universal Manifold Approximation Projection 2 (UMAP2) plots of the *Seurat* clusters as represented in A-B. **E**, Violin plots for the gene expression distribution of the vascular endothelium (top), mesenchymal cells (middle) scores (sum of log2 gene expression) and *HBEGF* (bottom, log2 expression level) in the *Seurat*  cell clusters as defined in A. **F**, Co-expression map between endothelial score (blue, sum of log2 gene expression) and *HBEGF* expression (orange, log2 expression) in the *Seurat*  cell clusters as defined in A-B. Co-expression is visualized in green. **G**, Co-expression map between endothelial score (blue, sum of log2 gene expression) and *HBEGF* expression (orange, log2 expression) in the *Seurat*  cell clusters as defined in A-B. Co-expression is visualized in green. **H**, *Seurat* Human GB cell clustering of UFK260 core GBM biopsy. Histological section stained with hematoxylin/eosin (H&E, pink/purple). **I**, *Seurat* cell labels from panel A as presented in panels C-D. **J**, vascular endothelium (top, includes *PECAM1*, *PODXL* and *COL4A1*) and mesenchymal-like (bottom, includes *CD44*, *VIM*, *P4HA1* and *HIF1A*) gene expression scores superimposed on the Universal Manifold Approximation Projection 2 (UMAP2) plots of the *Seurat* clusters as represented in A-B. Score expressed as sum of log2 gene expression. **K**, HBEGF gene expression (log2 expression) superimposed on the Universal Manifold Approximation Projection 2 (UMAP2) plots of the *Seurat* clusters as represented in A-B. **L**, Violin plots for the gene expression distribution of the vascular endothelium (top), mesenchymal cells (middle) scores (sum of log2 gene expression) and *HBEGF* (bottom, log2 expression level) in the *Seurat*  cell clusters as defined in A. **M**, Co-expression map between endothelial score (blue, sum of log2 gene expression) and *HBEGF* expression (orange, log2 expression) in the *Seurat*  cell clusters as defined in A-B. Co-expression is visualized in green. **N**, Co-expression map between endothelial score (blue, sum of log2 gene expression) and *HBEGF* expression (orange, log2 expression) in the *Seurat*  cell clusters as defined in A-B. Co-expression is visualized in green.


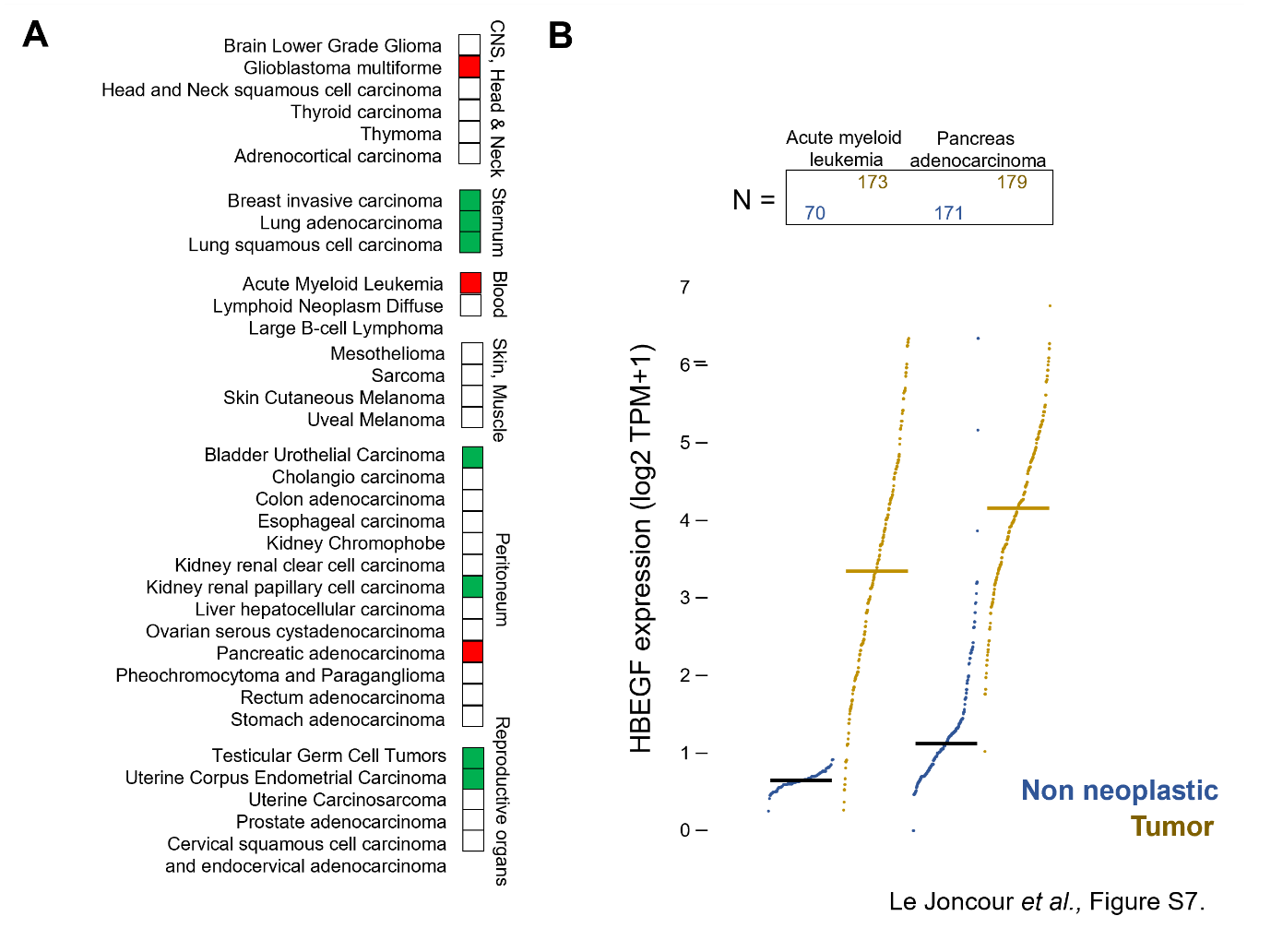


**Supplementary Figure S7: HPNPs target is upregulated in specific peripheral malignancies, enabling therapeutic potential of HPNP beyond CNS tumors**. **A,** HBEGF expression (green, downregulation, white, no change, red, upregulation) in neoplastic vs normal tissue from the TCGA (N = 9663) and non neoplastic tissue from GTEx (N = 5540). **B**, HBEGF gene expression (log2 TPM+1) in acute myeloid leukemia and pancreatic adenocarcinoma (tumor data from the TCGA and non neoplastic tissue from GTEx).

**Safety assessment in male and female rats using high and low dose of HP-NPs**

The safety of HP-NPs was established in eight-week old WIST rats (RccHan®:WIST). For this purpose, rats were exposed to intensive treatment regime to estimate the maximum tolerability/toxicity by daily injection of HP-NPs in either high dose (20 mg/kg) or low dose (2 mg/kg), in both male and female rats for 14 days (Supplementary Table S2). Experiments were carefully carried out to minimize bleeding by implementing slow rate infusions (>3 min) instead of injections (< 30s). The experiments were paused in high dose HP-NP group at some timepoints as indicated in the Supplement Table S3. Two males in the high dose group showed clinical signs of hemorrhage (injection d4 and d8, respectively) in the form of weakness. Interestingly, subcutaneous saline injections reversed the condition.

**Supplementary Table S2**: Body weight gain during the 14-day dosing study. The data were analyzed using a 1-way ANOVA approach, with dose group as the treatment factor and sex as a blocking factor. The response was rank transformed prior to analysis to stabilize the variance. Sex had an overall effect (p=0.0002) whereas Dose group had not (p=0.29).

| Sex | Group | *N* | Initial weight (g) | Final weight (g) | Body weight gain (g) |
| --- | --- | --- | --- | --- | --- |
| Females | High dose | 5 | 191 ± 7 | 226 ± 12 | 36 ± 6 |
|  | Low dose | 5 | 199 ± 9 | 228 ± 9 | 30 ± 6 |
|  | Placebo | 2 | 206 ± 15 | 233 ± 8 | 28 ± 6 |
| Males | High dose | 5 | 307 ± 18 | 352 ± 19 | 44 ± 7 |
|  | Low dose | 5 | 324 ± 30 | 366 ± 30 | 42 ± 7 |
|  | Placebo | 2 | 306 ± 37 | 352 ± 40 | 46 ± 4 |

**Supplementary Table S3**: Injection Scheme in rats treated for 14d with HP-NP injections.

**Supplementary Table S4.** Hematology in rats after 14-day intravenous injection of HP-NPs.

|  | |  | Females | | | Males | | |
| --- | --- | --- | --- | --- | --- | --- | --- | --- |
|  |  | Reference values* | Low dose (n=3-4) | High dose (n=4) | Placebo (n=1) | Low dose (n=5) | High dose (n=5) | Placebo (n=2) |
| Erythrogram | EPC (10^9^/L) | F 8.02 ± 0.53  M 8.39 ± 0.67 | 6.8 ± 0.17 | 6.2 ± 0.65 | 6.1 | 31.1 ± 53.1 | 6.7 ± 0.5 | 7.5 ± 0.14 |
|  | Hemoglobin (g/L) | F 152 ± 9  M 157 ± 10 | 125 ± 6 | 120 ± 9.7 | 113 | 109 ± 58 | 127 ± 12 | 139 ± 4 |
|  | Hematocrit (L/L) | F 0.43 ± 0.03  M 0.45 ± 0.03 | 0.38 ± 0.03 | 0.36 ± 0.03 | 0.33 | 0.41 ± 0.04 | 0.4 ± 0.02 | 0.41 ± 0.01 |
|  | MCV (fL) | F 53.8 ± 2.3  M 53.5 ± 2.4 | 55.3 ± 2.5 | 58.25 ± 1.26 | 54 | 56.4 ± 1.3 | 59 ± 1.8 | 55 ± 0 |
|  | MCHC (g/L) | F 353 ± 13  M 349 ± 12 | 334 ± 7.8 | 332 ± 5 | 343 | 327.8 ± 5.5 | 322.5 ± 13.5 | 335 ± 1.4 |
|  | Reticulocytes (x10^9^/L) | F: 217 ± 57  M: 238 ± 62 | 251 ± 37 | 341 ± 104 | 222 | 360 ± 49 | 394 ± 50 | 229 ± 59 |
| Leukogram | Leucocytes (10^9^/L) | F 3.12 ± 1.49  M 4.52 ± 1.81 | 3.8 ± 1.3 | 3.2 ± 1.1 | 2.7 | 5.76 ± 1.78 | 4.25 ± 2.32 | 6.20 ± 2.4 |
|  | Eosinophils (10e9/L) | F 0.05 ± 0.03  M 0.06 ± 0.04 | 0.1 | 0.15 ± 0.07 | <0.1 | 0.15 ± 0.06 | 0.1 | 0.1 |
|  | Lymphocytes (10^9^/L) | F 2.5 ± 1.21  M 3.63 ± 1.56 | 3.27 ± 0.99 | 2.38 ± 0.84 | 2.6 | 4.48 ± 1.17 | 3.45 ± 2.26 | 5.45 ± 2.19 |
|  | Monocytes (10^9^/L) | F 0.06 ± 0.03  M 0.08 ± 0.04 | 0.1 | 0.13 ± 0.06 | <0.1 | 0.14 ± 0.05 | 0.1 | <0.1 |
|  | Platelets (10^9^/L) | F 929 ± 133  M 904 ± 137 | 666 ± 567 | 755 ± 167 | 12 | 748 ± 141 | 653 ± 218 | 790 ± 59 |
| Coagulation | PT (s) | F: 13.4 ± 0.86  M: 13.6 ± 0.8 | 14.5 ± 0.4 | 14.3 ± 1 | Missing value | 16.4 ± 1 | 14.4 ± 0.8 | 15.1 ± 1 |
|  | PPT (s) | F. 20 ± 4.2  M: 21.6 ± 4.1 | 17.2 ± 2.7 | 16.2 ± 1.8 | Missing value | 17.4 ± 2.8 | 16 ± 0.4 | 15.2 ± 1.1 |

* Clinical Laboratory Parameters for CRL:WI (Han) in 8-16 w old rats, 2008

**Supplementary Table S5**: Clinical Chemistry in rats after 14-day intravenous injection of HP-NP in Serum (S) and Plasma (P).

|  |  | Females | | | Males | | |
| --- | --- | --- | --- | --- | --- | --- | --- |
| Serum (S)/Plasma (P) | Reference values* | Low dose (n=3) | High dose (n=4) | Placebo | Low dose (n=5) | High dose (n=4) | Placebo (n=2) |
| S/P-ALAT (µmol/L) | F: 0.42 ± 0.15  M: 0.47 ± 0.12 | 0.43 ± 0.15 | 0.60 ± 0.20 | Missing value | 0.44 ± 0.09 | 0.40 ± 0.08 | 0.55 ± 0.07 |
| S/P-Albumin (g/L) | F: 4.4 ± 0.5  M: 4 ± 0.4 | 34.00 ± 3.46 | 32.00 ± 0.82 | Missing value | 27.80 ± 1.48 | 27.75 ± 2.22 | 29.00 ± 1.41 |
| S/P-ASAT (µkat/L) | F: 1.7 ± 0.52  M: 1.75 ± 0.33 | 1.93 ± 0.65 | 1.95 ± 1.58 | Missing value | 1.04 ± 0.09 | 1.23 ± 0.46 | 1.15 ± 0.07 |
| S/P-Calcium (mmol/L) | F: 2.62 ± 0.1  M: 2.59 ± 0.12 | 2.60 ± 0.20) | 2.63 ± 0.21 | Missing value | 2.46 ± 0.44 | 2.68 ± 0.05 | 2.55 ± 0.07 |
| S/P-Potassium (mmol/L) | F: 4.07 ± 0.37  M: 4.48 ± 0.44 | 3.80 ± 0.35 | 3.98 ± 0.41 | Missing value | 5.22 ± 1.26 | 4.48 ± 0.64 | 4.30 ± 0.57 |
| S/P-Creatinine (µmol/L) | F: 35.36 ± 8.84  M: 26.52 ± 8.84 | 33.00 ± 1.73 | 31.25 ± 3.95 | Missing value | 27.00 ± 3.32 | 27.00 ± 3.16 | 28.50 ± 3.54 |
| S/P-Sodium (µmol/L) | F: 144 ± 2  M: 146 ± 2 | 138.33 ± 0.58 | 138.50 ± 2.38 | Missing value | 141.20 ± 1.64 | 140.00 ± 0.82 | 139.50 ± 2.12 |
| S/P-Urea (µmol/L) | F: 6.89 ± 1.32  M:6.11 ± 1.04 | 6.97 ± 0.46 | 6.58 ± 1.10 | Missing value | 6.74 ± 0.36 | 6.80 ± 0.68 | 6.65 ± 0.21 |
| S-Globulin (g/L) | F: 20 ± 2  M: 20 ± 2 | 28.00 ± 3.46 | 25.75 ± 1.89 | Missing value | 28.20 ± 2.39 | 27.25 ± 2.0 | 28.50 ± 3.54 |
| S-Protein (g/L) | F: 63 ± 5  M: 60 ± 6 | 62.00 ± 6.93 | 58.00 ± 2 | Missing value | 56.20 ± 3.83 | 55.50 ± 3.70 | 57.50 ± 4.95 |

* Clinical Laboratory Parameters for CRL:WI (Han) in 8-16 w old rats, 2008

**Supplementary Table S6**: Individual values from haematology in rats after 14d intravenous injection of HP-NP.

**Blood cell types**

| Animal | Dose | blood sample | sex | Eosinophils | EPC | EVF | HB | LPC | Lymphocytes | MCHC | MCV | Monocytes | Reticulocytes | Reticulocytes % | Segmented neutrophils | Thrombocytes |
| --- | --- | --- | --- | --- | --- | --- | --- | --- | --- | --- | --- | --- | --- | --- | --- | --- |
| 18 | 0.00 | no | m |  | 7.40 | 0.40 | 136.00 | 4.50 | 3.90 | 336.00 | 55.00 | 0.00 | 187.00 | 2.60 | 0.40 | 832.00 |
| 19 | 0.00 | no | m | 0.10 | 7.60 | 0.42 | 142.00 | 7.90 | 7.00 | 334.00 | 55.00 | 0.10 | 271.00 | 3.60 | 0.60 | 749.00 |
| 3 | 0.00 | no | f | 0.00 | 6.10 | 0.33 | 113.00 | 2.70 | 2.60 | 343.00 | 54.00 | 0.00 | 222.00 | 3.60 | 0.00 |  |
| 15 | 2.00 | no | m | 0.20 | 8.10 | 0.45 | 145.00 | 7.30 | 4.80 | 325.00 | 55.00 | 0.20 | 351.00 | 4.30 | 1.40 | 641.00 |
| 17 | 2.00 | no | m | 0.10 | 7.50 | 0.44 | 143.00 | 8.00 | 6.40 | 328.00 | 58.00 | 0.20 | 445.00 | 5.90 | 1.20 | 899.00 |
| 20 | 2.00 | no | m | 0.20 | 7.40 | 0.41 | 130.00 | 5.00 | 3.90 | 321.00 | 55.00 | 0.10 | 321.00 | 4.30 | 0.80 | 753.00 |
| 23 | 2.00 | yes | m | 0.00 | 7.00 | 0.38 | 126.00 | 4.00 | 3.60 | 336.00 | 57.00 | 0.10 | 340.00 | 5.10 | 0.40 | 874.00 |
| 24 | 2.00 | yes | m | 0.10 | 6.40 | 0.37 | 120.00 | 4.50 | 3.70 | 329.00 | 57.00 | 0.10 | 346.00 | 5.40 | 0.70 | 575.00 |
| 6 | 2.00 | yes | f | 0.00 | 6.90 | 0.38 | 127.00 | 3.20 | 2.60 | 332.00 | 55.00 | 0.00 | 232.00 | 3.30 | 0.50 | 971.00 |
| 7 | 2.00 | no | f | 0.00 | 6.60 | 0.35 | 119.00 | 2.90 | 2.80 | 343.00 | 53.00 | 0.00 | 229.00 | 3.50 | 0.00 |  |
| 9 | 2.00 | no | f | 0.10 | 6.90 | 0.40 | 130.00 | 5.30 | 4.40 | 328.00 | 58.00 | 0.10 | 294.00 | 4.30 | 0.60 | 1016.00 |
| 11 | 20.00 | no | f | 0.20 | 5.60 | 0.34 | 114.00 | 4.00 | 2.50 | 339.00 | 60.00 | 0.10 | 455.00 | 8.10 | 1.30 | 837.00 |
| 13 | 20.00 | yes | m | 0.10 | 6.00 | 0.37 | 111.00 | 3.00 | 2.00 | 303.00 | 61.00 | 0.10 | 462.00 | 7.70 | 0.70 | 840.00 |
| 14 | 20.00 | no | m | 0.00 | 7.20 | 0.41 | 135.00 | 4.50 | 4.00 | 328.00 | 57.00 | 0.10 | 400.00 | 5.60 | 0.30 | 831.00 |
| 16 | 20.00 | no | m | 0.10 | 6.80 | 0.41 | 136.00 | 7.40 | 6.40 | 334.00 | 60.00 | 0.10 | 346.00 | 5.10 | 0.70 | 544.00 |
| 2 | 20.00 | no | f | 0.00 | 5.70 | 0.33 | 110.00 | 1.60 | 1.20 | 333.00 | 58.00 | 0.00 | 227.00 | 4.00 | 0.30 | 506.00 |
| 22 | 20.00 | yes | m | 0.10 | 6.80 | 0.40 | 128.00 | 2.10 | 1.40 | 325.00 | 58.00 | 0.10 | 369.00 | 5.40 | 0.50 | 400.00 |
| 4 | 20.00 | no | f | 0.10 | 6.90 | 0.39 | 130.00 | 3.30 | 2.60 | 331.00 | 57.00 | 0.10 | 284.00 | 4.20 | 0.60 | 866.00 |
| 8 | 20.00 | no | f | 0.00 | 6.60 | 0.39 | 127.00 | 3.90 | 3.20 | 327.00 | 58.00 | 0.20 | 399.00 | 6.00 | 0.50 | 810.00 |

**Supplementary Table S7**: Individual values of clinical chemistry in rats after 14d intravenous injection of HP-NP, dosed in serum (S) and plasma (P).

| Animal | Dose | blood sample | sex | S/P-ALAT | S/P-Albumine | S/P-ASAT | S/P-Calcium | S/P-Potassium | S/P-Creatinine | S/P-Sodium | S/P-Urea | S-Globuline | S-Protein |
| --- | --- | --- | --- | --- | --- | --- | --- | --- | --- | --- | --- | --- | --- |
| 18 | 0.00 | no | m | 0.50 | 30.00 | 1.10 | 2.60 | 3.90 | 31.00 | 141.00 | 6.50 | 31.00 | 61.00 |
| 19 | 0.00 | no | m | 0.60 | 28.00 | 1.20 | 2.50 | 4.70 | 26.00 | 138.00 | 6.80 | 26.00 | 54.00 |
| 3 | 0.00 | no | f |  |  |  |  |  |  |  |  |  |  |
| 15 | 2.00 | no | m | 0.60 | 30.00 | 1.10 | 2.80 | 5.10 | 22.00 | 140.00 | 6.60 | 32.00 | 62.00 |
| 17 | 2.00 | no | m | 0.40 | 28.00 | 1.10 | 2.60 | 4.70 | 28.00 | 143.00 | 6.90 | 27.00 | 55.00 |
| 20 | 2.00 | no | m | 0.40 | 26.00 | 1.10 | 2.50 | 4.20 | 31.00 | 143.00 | 6.40 | 27.00 | 53.00 |
| 23 | 2.00 | yes | m | 0.40 | 28.00 | 0.90 | 1.70 | 7.40 | 28.00 | 140.00 | 6.50 | 29.00 | 58.00 |
| 24 | 2.00 | yes | m | 0.40 | 27.00 | 1.00 | 2.70 | 4.70 | 26.00 | 140.00 | 7.30 | 26.00 | 53.00 |
| 6 | 2.00 | yes | f | 0.30 | 32.00 | 1.30 | 2.40 | 4.20 | 32.00 | 138.00 | 6.70 | 26.00 | 58.00 |
| 7 | 2.00 | no | f | 0.40 | 32.00 | 2.60 | 2.60 | 3.60 | 32.00 | 138.00 | 6.70 | 26.00 | 58.00 |
| 9 | 2.00 | no | f | 0.60 | 38.00 | 1.90 | 2.80 | 3.60 | 35.00 | 139.00 | 7.50 | 32.00 | 70.00 |
| 11 | 20.00 | no | f | 0.50 | 32.00 | 1.40 | 2.40 | 4.50 | 28.00 | 136.00 | 5.80 | 23.00 | 55.00 |
| 13 | 20.00 | yes | m | 0.30 | 25.00 | 1.90 | 2.70 | 4.60 | 31.00 | 140.00 | 6.10 | 25.00 | 51.00 |
| 14 | 20.00 | no | m | 0.50 | 27.00 | 1.10 | 2.60 | 5.30 | 28.00 | 139.00 | 7.70 | 26.00 | 54.00 |
| 16 | 20.00 | no | m | 0.40 | 29.00 | 1.00 | 2.70 | 3.80 | 25.00 | 140.00 | 6.90 | 29.00 | 58.00 |
| 2 | 20.00 | no | f | 0.90 | 31.00 | 4.30 | 2.90 | 4.00 | 37.00 | 141.00 | 8.20 | 27.00 | 59.00 |
| 22 | 20.00 | yes | m | 0.40 | 30.00 | 0.90 | 2.70 | 4.20 | 24.00 | 141.00 | 6.50 | 29.00 | 59.00 |
| 4 | 20.00 | no | f | 0.50 | 32.00 | 1.10 | 2.60 | 3.50 | 30.00 | 137.00 | 6.20 | 27.00 | 59.00 |
| 8 | 20.00 | no | f | 0.50 | 33.00 | 1.00 | 2.60 | 3.90 | 30.00 | 140.00 | 6.10 | 26.00 | 59.00 |
