# Supplementary material for: Therapeutic Reprogramming of Glioblastoma Phenotypic States Using Multifunctional Heparin Nanoparticles
Source: Adv Sci (Weinh). 2025 Nov 3;13(3):e09590. doi: 10.1002/advs.202509590 (PMC12806396; doi:10.1002/advs.202509590)
Supplement: Supplementary file 2 — Supporting Information [file ADVS-13-e09590-s001.zip › Figure 6 Extended Data.pptx]

## Slide 1
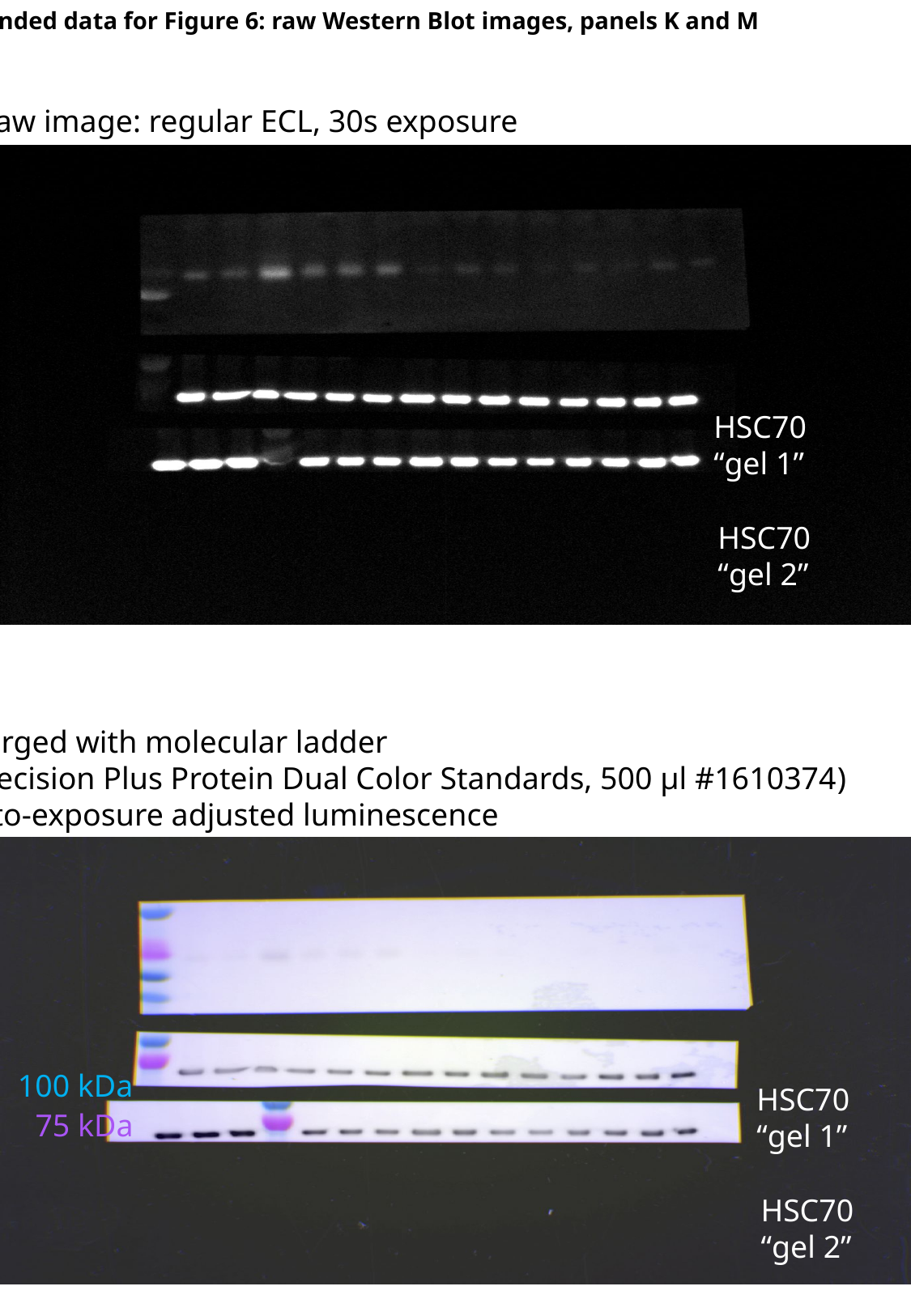

Extended data for Figure 6: raw Western Blot images, panels K and M
Raw image: regular ECL, 30s exposure
HSC70
“gel 1”
HSC70
“gel 2”
Merged with molecular ladder
(Precision Plus Protein Dual Color Standards, 500 µl #1610374)
auto-exposure adjusted luminescence
100 kDa
HSC70
“gel 1”
75 kDa
HSC70
“gel 2”

## Slide 2
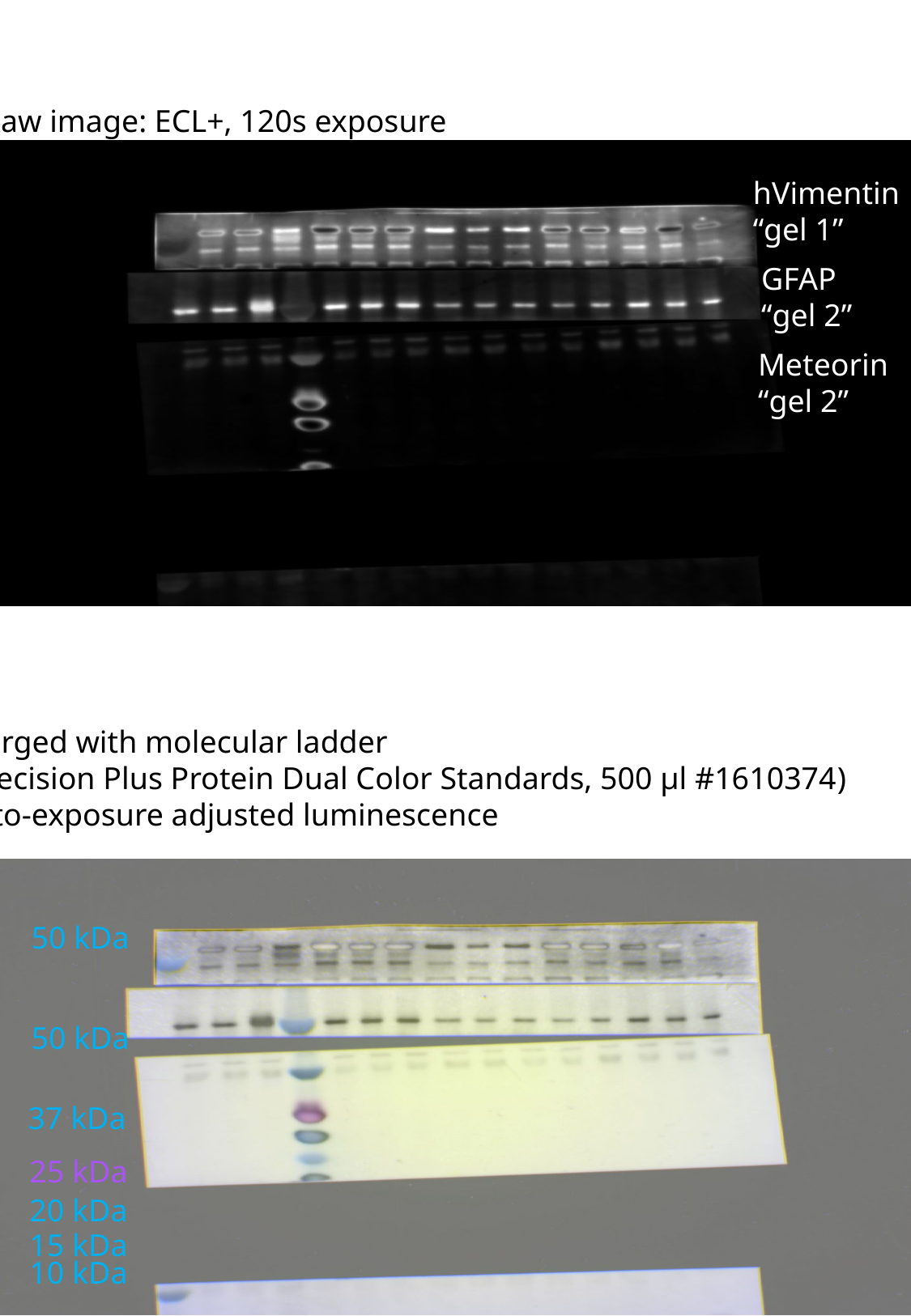

Raw image: ECL+, 120s exposure
hVimentin
“gel 1”
GFAP
“gel 2”
Meteorin
“gel 2”
Merged with molecular ladder
(Precision Plus Protein Dual Color Standards, 500 µl #1610374)
auto-exposure adjusted luminescence
50 kDa
50 kDa
37 kDa
25 kDa
20 kDa
15 kDa
10 kDa

## Slide 3
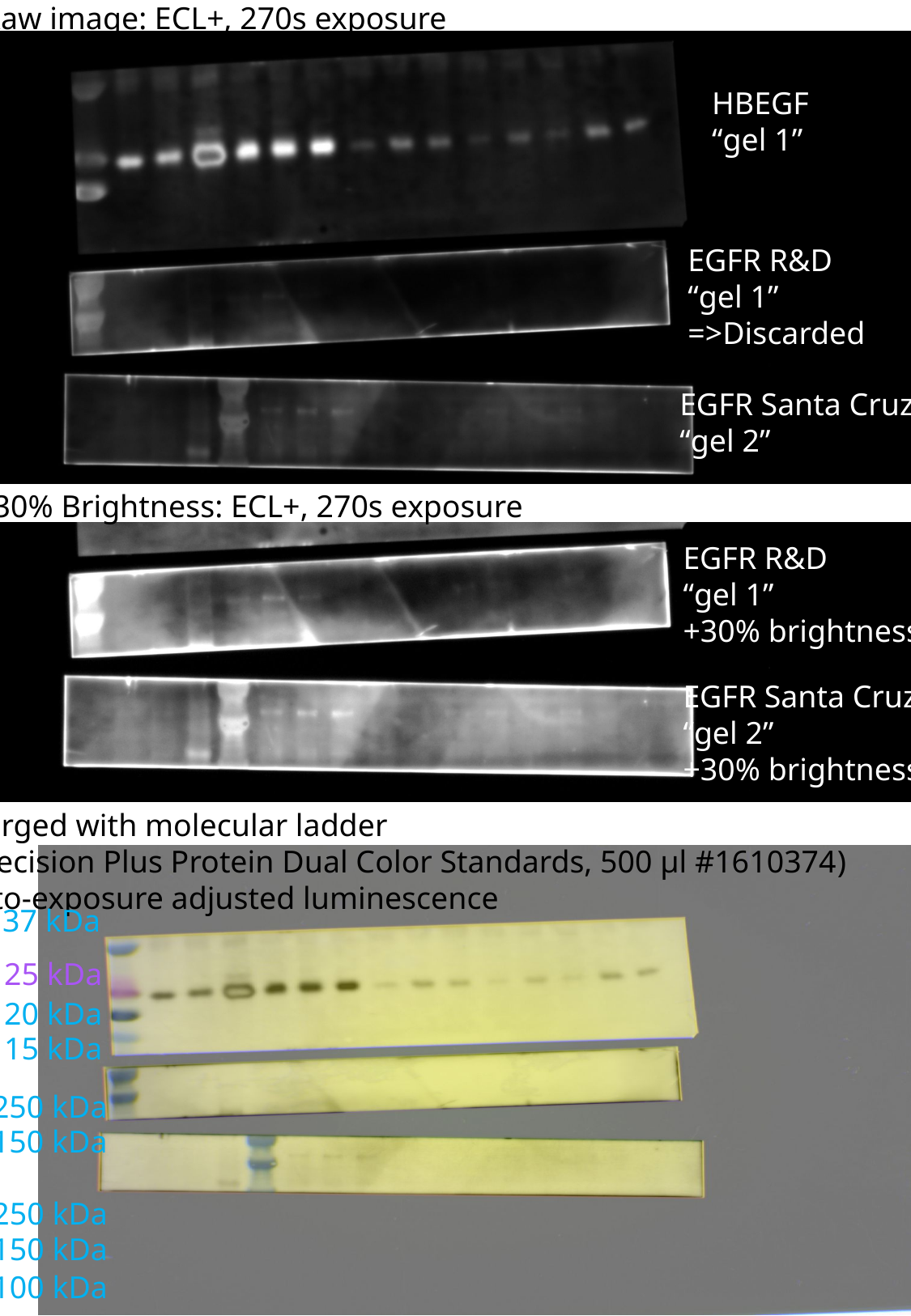

Raw image: ECL+, 270s exposure
HBEGF
“gel 1”
EGFR R&D
“gel 1”
=>Discarded
EGFR Santa Cruz
“gel 2”
+30% Brightness: ECL+, 270s exposure
EGFR R&D
“gel 1”
+30% brightness
EGFR Santa Cruz
“gel 2”
+30% brightness
Merged with molecular ladder
(Precision Plus Protein Dual Color Standards, 500 µl #1610374)
auto-exposure adjusted luminescence
37 kDa
25 kDa
20 kDa
15 kDa
250 kDa
150 kDa
250 kDa
150 kDa
100 kDa
